# The extracellular interactome of the human adenovirus family reveals diverse strategies for immunomodulation

**DOI:** 10.1038/ncomms11473

**Published:** 2016-05-05

**Authors:** Nadia Martinez-Martin, Sree R. Ramani, Jason A. Hackney, Irene Tom, Bernd J. Wranik, Michelle Chan, Johnny Wu, Maciej T. Paluch, Kentaro Takeda, Philip E. Hass, Hilary Clark, Lino C. Gonzalez

**Affiliations:** 1 Department of Protein Chemistry, Genentech, 470 East Grand Avenue, South San Francisco, California 94080, USA; 2 Department of Bioinformatics and Computational Biology, Genentech, 455 East Grand Avenue, South San Francisco, California 94080, USA

## Abstract

Viruses encode secreted and cell-surface expressed proteins essential to modulate host immune defenses and establish productive infections. However, to date there has been no systematic study of the extracellular interactome of any human virus. Here we utilize the E3 proteins, diverse and rapidly evolving transmembrane-containing proteins encoded by human adenoviruses, as a model system to survey the extracellular immunomodulatory landscape. From a large-scale protein interaction screen against a microarray of more than 1,500 human proteins, we find and validate 51 previously unidentified virus–host interactions. Our results uncover conserved strategies as well as substantial diversity and multifunctionality in host targeting within and between viral species. Prominent modulation of the leukocyte immunoglobulin-like and signalling lymphocyte activation molecule families and a number of inhibitory receptors were identified as hubs for viral perturbation, suggesting unrecognized immunoregulatory strategies. We describe a virus–host extracellular interaction map of unprecedented scale that provides new insights into viral immunomodulation.

## Abstract (web summary)

Viruses interact with their hosts via secreted and membrane-bound proteins to affect host immune responses and virulence. Here the authors contribute to our understanding of this relationship with an extracellular interaction map of human and adenoviral E3 immunomodulatory proteins.

## Graphical abstract

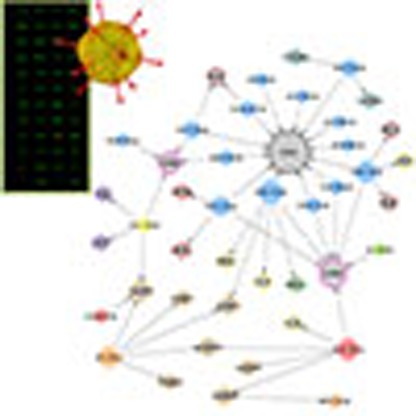

Viruses must evade or modulate the immune response to infect, spread and finally persist in the host. As a consequence of the pressure imposed by the immune system in the context of restricted size genomes, viruses have evolved optimized immunomodulators that efficiently regulate a variety of host processes. Characterizing how viruses interact with the immune network is essential to understand viral pathogenesis and may lead to new approaches for treating human diseases. Proteomic studies of virus–host interactions have provided insights into host biology and viral-induced disease^1^,^2^,^3^, as well as the discovery of new factors involved in immune processes^4^. Nevertheless, interactions with host extracellular molecules, including secreted or plasma membrane-expressed proteins, are highly underrepresented in previous data sets. Here we utilize a protein microarray platform, containing more than 1,500 extracellular human proteins (∼50% of all predicted secreted and plasma membrane-expressed single-transmembrane domain genes)^5^,^6^,^7^, to gain functional insights and evaluate the diversity of virus–host interactions across a comprehensive set of candidate immunomodulatory cell-surface expressed proteins encoded by human adenoviruses (HAdVs).

HAdVs, which comprise over 70 types divided into seven species (A to G), establish acute and persistent infections in a number of tissues^8^. Despite the increasing importance of HAdVs as both emerging pathogens and therapeutic vectors^9^,^10^, information on the interaction of these viruses with the host immune system is surprisingly scarce^11^. Most HAdV species-specific genes are concentrated in the early transcription unit 3 (E3), comprising five to nine genes that are dispensable for viral entry and replication^11^. The ectodomains (ECDs) of the E3 proteins display substantial diversity in amino acid sequence across and within HAdV species and constitute one of the most divergent elements within the genome^12^,^13^. Despite the variable outcomes, most species predominantly utilize common and broadly expressed entry receptors^14^, and therefore differential immunoregulatory functions exerted by the E3 proteins are probably important contributors to species-specific pathogenesis^11^. Here we take advantage of such unique intrinsic variability to evaluate the effect of viral diversity in extracellular host targeting and gain functional insights into novel immunoregulatory strategies exploited by HAdVs.

We present the first large-scale study of the extracellular interactome of a complete family of human viruses. This study provides the largest host–pathogen extracellular network to date, as well as the only comprehensive analysis of HAdV E3 immunoregulatory proteins, identifying 51 new virus–host interactions encompassing five viral species. Our data reveal diversity in extracellular targeting of immune receptors as well as conserved strategies within and between viral species. Further, these findings identify viral targets that may be involved in unrecognized mechanisms of immunoregulation, such as the modulation of inhibitory immune receptors. These results demonstrate that HAdVs have evolved complex multifunctional proteins to manipulate diverse host cell-surface receptors, a strategy probably utilized by other human viruses and a possible mechanism contributing to the differential HAdV disease.

## Results

### HAdVs to survey the extracellular immunomodulatory landscape

**Figure 1:**
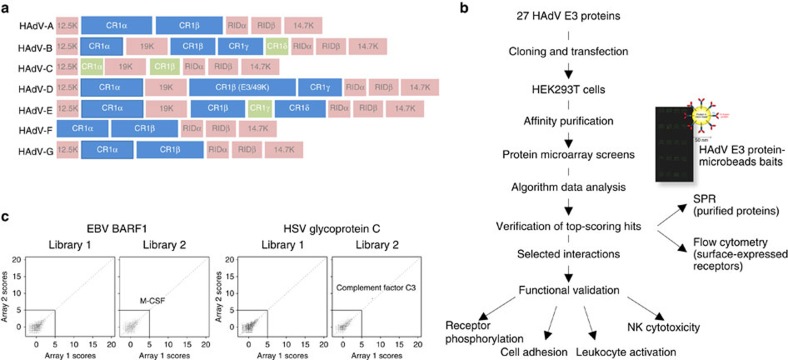
HAdVs as a model to study the extracellular interactome. (**a**) Schematic of the E3 transcription unit for each HAdV species, adapted with permission obtained from Elsevier Ltd^60^. Genes showing higher relative conservation are represented by pink boxes, whereas green colour indicates species-specific genes predicted to encode for intracellular products. Blue boxes represent divergent genes predicted to encode for extracellular proteins included in this study. (**b**) Summary of the workflow for the discovery of the HAdV-host extracellular interaction network. (**c**) Intersection plots representing screen results obtained for proof-of-concept assays. Each viral protein was screened against two extracellular human protein libraries, representing 1,555 genes. Each scatter plot represents two replicate microarray data sets (Array 1 and Array 2), where dots represent average scores for each protein in the library. The lower left square in each plot represents an empirically set cutoff of 5 and contains all non-hit proteins. The data analysis and scoring procedures are described in the ‘Methods’ section. M-CSF was identified as a hit for EBV BARF1 (left plot), whereas HSV glycoprotein C targeted the complement factor C3 (right plot).

The HAdV E3 region exhibits substantial sequence diversity and includes a variable number of ORFs as well as genes uniquely encoded by particular species^12^ (Fig. 1a and Supplementary Fig. 1a). With a few exceptions, previous studies on the roles of E3 proteins focused on species C, which consists of the genus-conserved proteins (12.5K, 19K, RIDα, RIDβ and 14.7K) and two species-specific proteins (CR1α and CR1β) that encode for intracellular products^11^. By contrast, a number of E3 proteins encoded by other HAdV species are predicted to contain a signal peptide and a transmembrane domain, suggesting functions in the extracellular environment (Supplementary Fig. 1b). Here we sought to understand the effect of such viral immunomodulatory protein diversity in the interaction with the host from a global perspective. To accomplish this, all HAdV E3 genes predicted to encode for extracellular products (Fig. 1a) were cloned, and the resultant purified proteins screened against an unbiased, functional protein microarray platform previously developed and validated in our lab that consists of over 1,500 secreted or transmembrane-containing human proteins^6^ (Supplementary Table 1). The virus–host candidate interactions found in these high throughput screens were validated by orthogonal methods, followed by functional analysis of selected interactions (Fig. 1b). Initial proof-of-concept screens successfully identified known virus–host interactions (Fig. 1c).

### HAdV extracellular interactome

**Figure 2:**
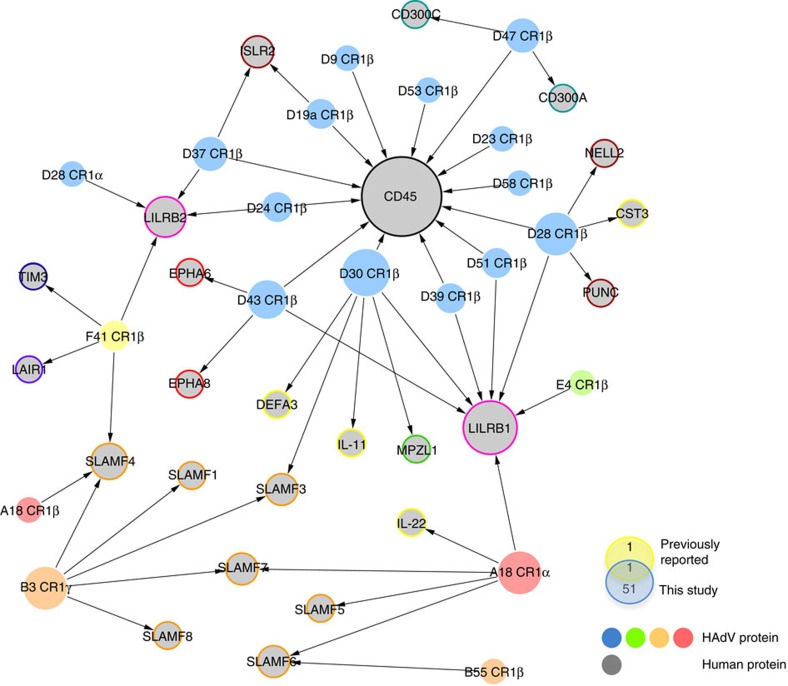
HAdV E3 protein extracellular immunomodulatory interaction network. Network plot representing the new 51 virus–host interactions identified and experimentally validated from over 30,000 pairs analysed. Coloured filled nodes indicate HAdV species. Grey nodes represent host proteins grouped into different categories (orange, SLAMFs; pink, LIL; brown, nervous factors; red, ephrin receptors; cyan, CD300 family; yellow, secreted factors; green, MPLZ1; blue, TIM3 and purple, LAIR1). Node size reflects the number of interactions identified for each protein. Plot was generated using Cytoscape software. Diagram shows overlap between our network and previously characterized E3 protein–host factor extracellular interactions^16^.

To comprehensively analyse the landscape of extracellular interactions between the host and the HAdV E3 immunomodulatory proteins, we cloned and purified 27 E3 proteins encoded by six viral species, including HAdV-A, -B, -D, -E, -F and -G CR1α and CR1β genes; HAdV-B and D CR1γ and HAdV-E CR1δ genes. After exhaustive screening of all E3 proteins against the protein microarray libraries followed by statistical hit calling, we characterized a HAdV-host extracellular network comprising 51 new interactions among 20 viral proteins encoded by five HAdV species and 24 human factors (Fig. 2 and Supplementary Fig. 2. Raw data from the microarray screens are available in Supplementary Data 1 and have been deposited in the GEO database^15^). This study unequivocally supports the long suspected but experimentally unverified role of the E3 proteins in immunoregulation. We found that E3 protein diversity within and between HAdV species imparted significant variability in the interaction with the immune system. However, interestingly, conserved themes were observed across the HAdV family. In particular, widespread targeting of the leukocyte immunoglobulin-like (LIL) and the signalling lymphocyte activation molecule (SLAM) receptor families was identified. Furthermore, known or predicted inhibitory receptors were identified as prominent targets for most species. These findings point towards these receptor types as unrecognized hubs for viral modulation, suggesting novel strategies of immunoevasion. In addition, individual E3 proteins interacted with multiple host targets, likely conferring these proteins the ability to interfere with diverse host processes.

**Figure 3:**
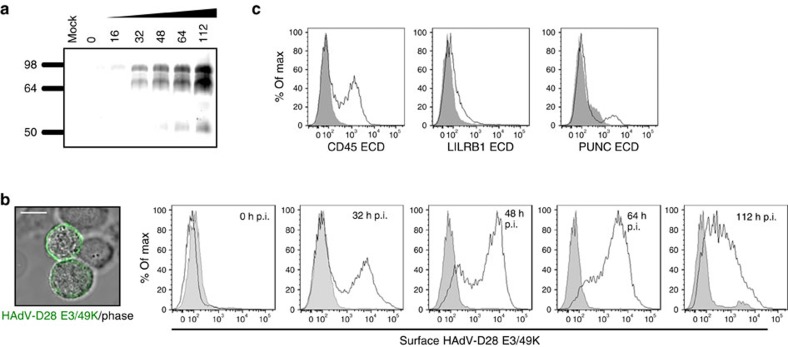
E3/49K is secreted and expressed on the surface of infected cells. (**a**) Analysis of E3/49K secretion from HAdV-D28-infected cells. Molecular sizes are indicated in kDa. Full immunoblot is shown in Supplementary Fig. 8a. (**b**) Detection of the E3/49K protein expressed at the surface of HAdV-D28-infected cells by immunofluorescence (left panel) and flow cytometry (right histograms). Grey-filled histograms represent staining corresponding to non-infected cells, black histograms show E3/49K surface expression on HAdV-D28-infected cells at the indicated hours post infection (p.i.). Scale bar, 10 μm. (**c**) Binding of CD45, LILRB1 and PUNC purified ECDs, found to interact with HAdV-D28 E3/49K, to infected cells. Grey-filled histograms represent binding of a control recombinant protein, whereas black histograms show binding of the indicated E3/49K hits to the surface of infected cells. Assays shown are representative of two independent experiments.

Species D HAdVs encodes the largest species-specific protein (E3/49K), characterized by significant intra-species variability (Supplementary Fig. 3), therefore providing a particularly suitable model to study the effect of viral diversity in host immune targeting. Notably, the E3/49K protein encoded by HAdV-D19a was recently found to target and modulate the surface phosphatase CD45 (ref. 16). To address the effect of such striking diversity on immune factor targeting, the E3/49K protein encoded by 13 different viral types, selected based on sequence variability, was included in the study. Interestingly, we found that despite the significant sequence diversity, all E3/49K proteins analysed targeted CD45 with exceptionally high affinity (Fig. 2, Supplementary Figs 2 and 4b). Moreover, selected E3/49K proteins bound additional host factors in a type-specific manner. Secretion of the E3/49K protein encoded by HAdV-D19a has been recently hypothesized to influence the progression of epidemic keratoconjunctivitis (EKC)^16^, disease commonly associated to certain viral types, including HAdV-D8, -D19a, -D37, along with the more recently sequenced types D53, D54 and D56. We addressed the cellular location of the E3/49K encoded by HAdV-D28, which considerably differs in sequence compared with its HAdV-D19a counterpart. Similar to the HAdV-D19a homologue, HAdV-D28 E3/49K was also detected in supernatants from infected cells (Fig. 3a). In addition, the viral protein was expressed on the cell surface (Fig. 3b), where it was able to bind to the HAdV-D28 E3/49K-specific host receptors identified, analysed as soluble ECDs (Fig. 3c). These results rule out the possibility that secretion of HAdV-D19a E3/49K is uniquely responsible for the development of EKC, and suggest that species D E3/49K proteins may in general impact immune functions both in infected and non-infected cells.

The specificity of all E3–host interactions was verified by surface plasmon resonance (SPR) using purified ECDs for each of the interacting partners (Supplementary Fig. 4) as well as by flow cytometry, utilizing HEK293T cells transiently expressing full-length candidate receptors for each E3 protein (Supplementary Fig. 5). The use of these orthogonal methods enabled us to verify most of the candidate hits identified from over 30,000 virus–host interactions pairs tested, therefore generating a fully validated interaction network. Nevertheless, given that our libraries represent around 50% of the human extracellular proteome, it is possible that certain E3 proteins interact with additional receptors that are not included in the microarray.

### Diverse functional targeting of the SLAM family

**Figure 4:**
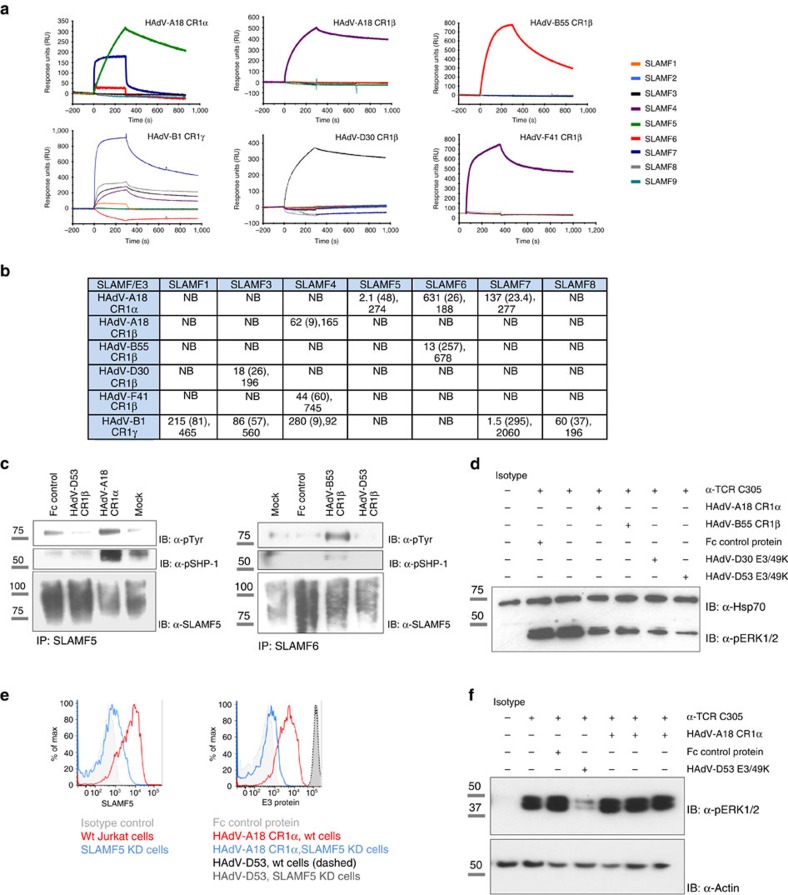
SLAM receptor family is a hub for E3-mediated interference. (**a**) Analysis of SLAM receptor ECDs (400 nM) binding to the E3 proteins indicated, immobilized on SPR chips. (**b**) Kinetic parameters calculated for the interaction between the E3 proteins and the SLAM receptors targeted. Binding affinities are shown as *K*_D_ (nM) values. The *χ*^2^, in parenthesis, followed by and *R*_max_ values indicate the goodness of the experimental fitting. NB, no binding. (**c**) HEL cells were stimulated as indicated and cell lysates were immunoprecipitated for SLAMF5 (left) or SLAMF6 (right), and immunoblotted to detect phosphorylation of tyrosine residues, SLAMF5, SLAMF6 or phosphoSHP-1. An irrelevant protein carrying the same tag (human Fc) as the viral proteins, named ‘Fc control’, was included in all the experiments throughout the manuscript to control for potential nonspecific effects due to the presence of this tag. (**d**) Jurkat cells were pre-incubated with control or E3 proteins before stimulation with an anti-TCR antibody and phosphorylation of ERK1/2 was analysed by immunoblotting. (**e**) Expression of SLAMF5 on the surface of wt or SLAMF5 knockdown (KD) Jurkat cells (left histogram) and binding of the indicated viral proteins to both cell types (right histogram). HAdV-D53 E3/49K binds to CD45 and was used as a control. (**f**) SLAMF5 KD cells were stimulated as in **d** to analyse the implication of this receptor in HAdV-A18 CR1α-mediated inhibition. The HAdV-A18 CR1α protein was assayed at 1, 10 and 50 nM (from left to right), whereas control proteins were added at 50 nM. Assays are representative of at least three independent experiments. Molecular masses are indicated in kDa. Full immunoblots are shown in Supplementary Fig. 8b–e.

Directed by our findings that different SLAM receptors were targeted by disparate E3 proteins, and since not all SLAM members were included in the protein microarrays, we used flow cytometry and SPR to analyse SLAM family binding specificity for all E3 proteins (Fig. 4a and Supplementary Fig. 6). SLAM receptors exert multiple functions in the immune response, including viral specific immunity^17^, but have not been implicated in HAdV infection. Interestingly, our results demonstrated that most HAdV species encoded at least one E3 protein that interacted with the SLAM receptors. Notably, the species A CR1α protein bound to three SLAM members, whereas the HAdV-B CR1γ protein interacted with five receptors, with different relative affinities (Fig. 4b). SLAM receptors are characterized by one or more intracellular tyrosine-based switch motifs that serve as docking sites for inhibitory signalling molecules upon receptor activation and subsequent phosphorylation^18^. We next investigated SLAMF5 and SLAMF6 activation upon leukocyte stimulation with the HAdV-A CR1α and HAdV-B CR1β proteins, which targeted SLAMF5 and SLAMF6 with high affinity, respectively. The E3 proteins stimulated SLAM activation, as detected by the increased phosphorylation observed in receptor immunoprecipitates from HEL cells (Fig. 4c), which we found to express high endogenous levels of both SLAM receptors (not shown). As a further control, we used the E3/49K protein encoded by HAdV-D53 that did not interact with any of the SLAM members. Receptor activation was accompanied by increased association to SHP-1, previously shown to mediate augmented negative signals^19^. Moreover, E3-mediated overactivation of the SLAMF receptors correlated with downregulation of TCR signalling in Jurkat cells, as shown by the reduced activation of ERK1/2 upon pre-incubation with HAdV-A CR1α or HAdV-B CR1β proteins (Fig. 4d). Further, the analysis of SLAMF5 knockdown Jurkat cells demonstrated that HAdV-A CR1α-driven inhibition of T-cell signalling was primarily mediated by SLAMF5 engagement, pointing towards a relevant role of this receptor in T-cell activation (Fig. 4e,f). Interestingly, we detected similar binding of the HAdV-B55 CR1β protein regardless of the expression of SLAMF6 on the cell surface (not shown); therefore, in this case, we cannot rule out that interaction with an unknown receptor(s) plays a role in the effect on T-cell signalling observed. Pre-incubation with HAdV-D30 and D53 E3/49K proteins potently downregulated TCR-mediated activation of the Jurkat cells, likely through CD45 abundantly expressed on the surface and in agreement with previous findings^16^.

### LILRB1 and LILRB2 are conserved targets

Similar to SLAM receptors, the LILR family comprises a set of related inhibitory and activating receptors that play relevant functions during infection^20^. The microarray screens identified LILRB1 as a top-scoring hit for five different E3/49K proteins (Supplementary Fig. 2). To better understand the interaction with the LILR family, we analysed binding of all E3 proteins to cells expressing 10 different LILR members characterized by activating or inhibitory functions^20^ (Supplementary Fig. 7). Notably, the E3/49K proteins encoded by HAdV-D24 and -D37 specifically targeted the related inhibitory receptor LILRB2, not present in the initial protein microarray screens. Moreover, species E CR1β and species A CR1α proteins targeted LILRB1, whereas species F CR1β and species D CR1α proteins bound LILRB2, results that were confirmed by SPR (Supplementary Fig. 7d).

**Figure 5:**
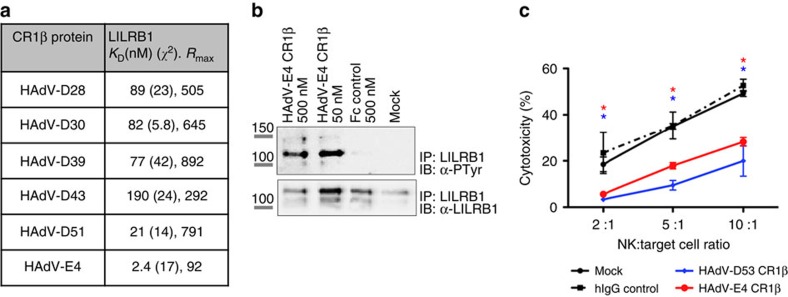
The inhibitory receptor LILRB1 is a semi-conserved, functional target. (**a**) Kinetic parameters calculated for the interaction between the E3 proteins and LILRB1 recombinant ECD. The *χ*^2^ and *R*_max_ values indicate the goodness of the experimental fitting. (**b**) PBMCs were stimulated as indicated and the activation of LILRB1 was analysed in receptor immunoprecipitates using an anti-phosphotyrosine antibody. Blots were reblotted against LILRB1 to control for similar protein loading. Molecular sizes are indicated in kDa. Full immunoblot is shown in Supplementary Fig. 8f. (**c**) K562 target cells were exposed to primary NK cells at the indicated effector:target ratios in the absence (mock) or presence of the indicated E3 proteins (100 nM). HAdV-D53 E3/49K significantly reduced NK-mediated cytotoxicity through interference with CD45 signalling, similar to the results described for HAdV-D19 E3/49K (ref. 16). HAdV-E4 CR1β inhibited NK cell killing (*P<0.01 (analysis of variance)). Assays are representative of at least two independent assays run in triplicates. Error bars represent s.d.

Next, the effect of the HAdV-E CR1β protein, which bound LILRB1 with the strongest affinity (Fig. 5a), was analysed. LILRB1 is an immunoreceptor tyrosine-based inhibitory motif (ITIM)-containing molecule that exerts a potent inhibitory function upon interaction with a broad range of MHC class I molecules and subsequent phosphorylation^20^. We found that the viral protein significantly increased tyrosine phosphorylation of LILRB1 immunoprecipitated from peripheral blood mononuclear cell lysates (Fig. 5b). Moreover, stimulation with the HAdV-E CR1 β protein impaired NK-mediated killing of the susceptible cell line K562 at all effector:target cell ratios analysed (Fig. 5c), possibly through targeting and activation of LILRB1 on the NK.

### Virus–host interaction discovery for functional prediction

**Figure 6:**
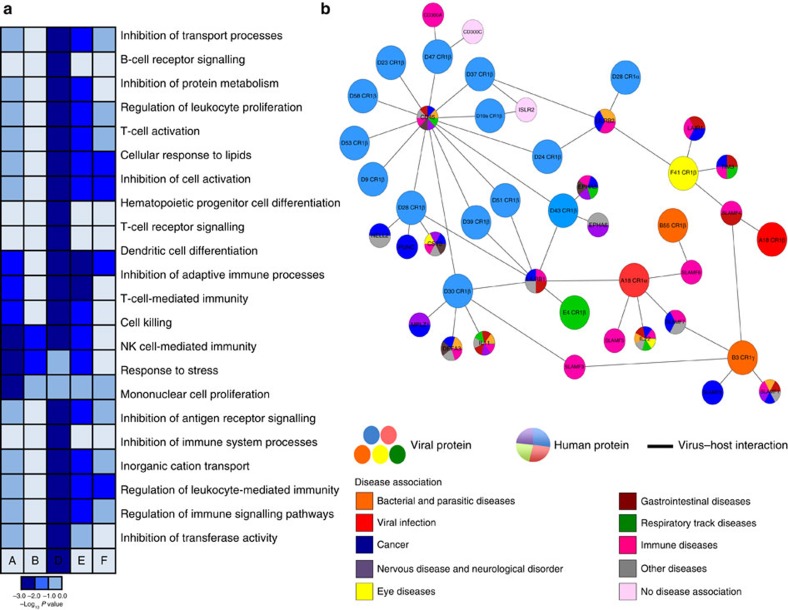
Gene ontology and disease association for the HAdV extracellular network. (**a**) Heat map depicting manually curated enriched biological processes of the host factors targeted by the HAdV E3 proteins obtained by a GO term analysis. Host genes were split by the viral species with which they interacted. These lists were separately queried for GO enrichment. The values represent the −log_10_ *P* value of the enrichment, indicated by different colours as shown in the accompanying scale. Grey colour denotes absence of genes in a particular GO category. (**b**) Overlaid of the E3 protein–host interaction network with disease-associated host proteins. HAdV E3 proteins are shown as colour nodes, edges indicate the virus–host interactions identified and pie charts represent the targeted host factors, which were coloured according to the association of each protein with disease. Disease terms from the DisGeNET database^22^ were manually collapsed to create curated terms for the extracellular HAdV network (colours are specified in the legend).

A main goal of this study was to utilize these virus–host interaction data to predict protein functions and identify new host factors involved in immune responses. We performed enrichment tests for gene ontology (GO) terms associated with the set of interactions identified for the HAdV E3 proteins (Fig. 6a and Supplementary Table 2). Known functions, such as the implication of LILRB1 in dendritic cell differentiation were accurately identified^21^. Further, these analyses allowed us to associate each viral species with certain immune pathways, suggesting a role for these processes in species-specific immunity. Next, we looked at the association of the E3-targeted host proteins with disease, using the DisGeNET database^22^ (Fig. 6b and Supplementary Table 3). Notably, most of these host factors had not been previously associated with viral infection, further highlighting the usefulness of our study to identify new molecules involved in antiviral responses. This correlation suggests that E3 protein-mediated interference with the functions of the host molecules identified may contribute to the disease caused by the different HAdV species.

### Adhesion receptors are modulated by HAdVs

**Figure 7:**
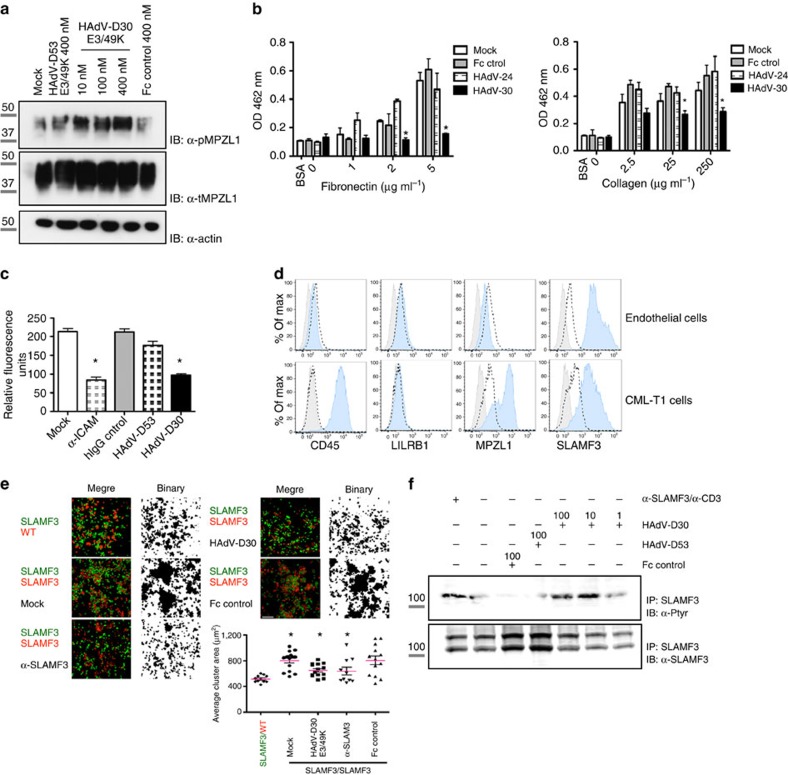
HAdV-D30 E3/49K modulates cell adhesion through MPZL1 and SLAMF3 targeting. (**a**) HeLa cells were stimulated with the indicated proteins (200 nM) and MPZL1 activation was analysed using an anti phospho-MPZL1 antibody. Full immunoblot is shown in Supplementary Fig. 8g. (**b**) HeLa cells were pre-stimulated with the MPLZ1 binder HAdV-D30 E3/49K or control proteins and incubated with (left) FN- or (right) collagen-coated plates and adhered cells were quantified. HAdV-D30 E3/49K blocks HeLa cell adhesion (*P<0.01, *P<0.001 and *P<0.05; *P<0.001, respectively, (analysis of variance)). Plot shows one representative experiment out of at least three independent assays run in triplicates. Mean±s.d. (**c**) Analysis of CML-T1 T cell adhesion to HUVEC endothelial cells. T cells were labelled with Calcein AM and incubated with the HUVEC monolayers during 30 min, after pre-incubation with the indicated proteins (100 nM). Cell adhesion was quantified using a fluorescence plate reader. Mean±s.d., triplicate data points from two to three independent experiments. HAdV-D30 E3/49K decreases T-cell adhesion to HUVEC cells (Students *t*-test, *P=0.0004 and *P=0.002, respectively). Scale bars=20 μm. (**d**) Expression of HAdV-D30 E3/49K identified receptors on the surface of HUVEC and CML-T1 cells. Grey-filled histograms represent unlabelled cells, dashed histograms show isotype antibody binding and blue histograms represent surface expression of the indicated molecules. (**e**) CHO cells transfected with SLAMF3 were stained with green or red dyes and co-incubated after pre-incubation with the indicated proteins. Cell cluster formation was monitored using a confocal microscope. Plot shows the quantification of one representative assay. HAdV-D30 E3/49K competes SLAMF3-mediated cell clustering (Students *t*-test, *P=0.0031, *P=0.035 and *P=0.025, respectively). Scale bar, 150 μm. (**f**) SLAMF3 activation was analysed in receptor immunoprecipitates. Assays are representative of at least two independent experiments. Molecular sizes are indicated in kDa. Full immunoblot is shown in Supplementary Fig. 8h.

One of the most interesting observations was the ability of most of the viral proteins to target multiple host receptors, and in particular, the propensity to engage receptors with known or predicted inhibitory functions. We analysed the functional effect of HAdV-D30 E3/49K, which interacted with CD45 and three receptors associated with inhibitory processes, LILRB1, SLAMF3 and MPLZ1. In accordance to its strong interaction with CD45, the viral protein potently inhibited T and NK activation *in vitro* (Fig. 4d not shown). MPZL1 is an Ig-domain receptor that contains two ITIMs and has been implicated in fibronectin-dependent migration and signalling and tumour metastasis^23^,^24^. We therefore tested whether HAdV-D30 would impact adhesion of MPZL1-expressing cells. Interestingly, the viral protein triggered activation of MPLZ1 in HeLa cells that endogenously express high levels of this receptor (Fig. 7a), and this correlated with a significant inhibition of cell adhesion to plates coated with the extracellular matrix proteins FN or collagen (Fig. 7b). We hypothesized that the E3/49K protein could also interfere with immune cell adhesion to endothelial monolayers. In fact, the viral protein abrogated T-cell attachment to endothelial cells (Fig. 7c). Interestingly, we detected significant levels of SLAMF3 on the surface of both the CML-T1 T cells and endothelial HUVEC cells assayed (Fig. 7d). Of note, SLAMF3 expression is not restricted to immune cell but was recently detected in hepatocytes, where it plays a role in Hepatitis C virus entry^25^ as well as in tumour cell progression^26^. SLAMF3-mediated homotypic contacts trigger the phosphorylation of intracellular tyrosine-based switch motifs and a series of inhibitory events^27^. Receptor targeting by the HAdV-D30 E3/49K protein significantly diminished SLAMF3-mediated formation of cell clusters (Fig. 7e), and modulated activation of the receptor in T cells (Fig. 7f). The HAdV-D53 E3/49K protein, which binds to CD45 but not SLAMF3, did not modify receptor activation nor alter T-cell adhesion to endothelial cells. These data further indicate that the HAdV E3 proteins have evolved to target and activate selected inhibitory receptors. Moreover, it suggests previously unrecognized functions of MPZL1 and SLAMF3 receptors in immune and endothelial cell communication, an axis that may be exploited by other viruses for immunoevasion.

**Figure 8:**
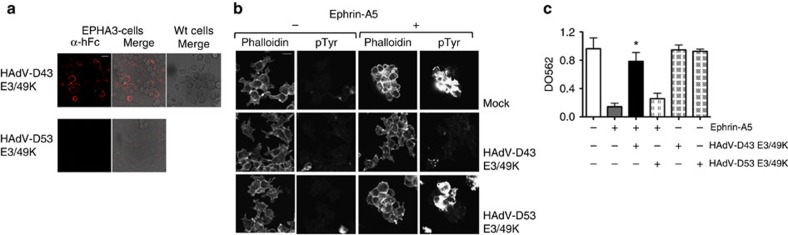
The E3/49K proteins modulate targeted receptor function. (**a**) Binding of HAdV-D43 and -D53 E3/49K proteins (100 nM) to wt (right) EPHA3-transfected (left) cells visualized by immunofluorescence. (**b**) Actin cytoskeleton and tyrosine phosphorylation changes upon stimulation with ephrin-A5, the viral proteins indicated or the combination of both. Assay shown is representative of two independent experiments. (**c**) Effects on adhesion of HEK293T cells to FN-coated plates. The cells were mock-treated or pre-incubated with the indicated viral proteins (100 nM), stimulated with Ephrin-A5 and incubated on FN-coated plates. Plots represents one assay run in quadruplicates out of two independent experiments. HAdV-D43 E3/49K protein counteracts ephrin-A5 effect on cell adhesion (Students *t*-test, (*P*<0.0001). Mean±s.d. is represented in all cases. Scale bars, 20 μm.

Ephrin receptors (EPHs) are the largest family of receptor tyrosine kinases (RTK) and play many essential functions in multiple processes^28^, including viral infections^29^,^30^. Interaction of EPHA3 with its ligand ephrin-A5 has been shown to modulate cell adhesion^31^,^32^. We found that HAdV-D43 E3/49K bound to EPHA3-expressing HEK cells (Fig. 8a). As previously described, stimulation with ephrin-A5 induced cell activation, rounding and retraction of cell processes (Fig. 8b), leading to reduced adhesion to FN (Fig. 8c)^31^. On the contrary, pre-incubation with the HAdV-D43 protein counteracted ephrin-A5-mediated effect on cell spreading and adhesion (Fig. 8b,c), suggesting that the viral protein impacts ephrin-A5 binding to its receptor. These results confirm the feasibility of our approach to detect functional viral protein–host receptor interactions and further implicate HAdVs interference with adhesion processes, a mechanism that might play a subversive role in host immune control.

A summary of the novel E3 protein–host interactions identified, screening and validation methodologies utilized, as well as the functional effects observed is shown in Table 1.

## Discussion

Our data reveal that through gene diversification HAdVs have developed a broad targeted landscape for immunomodulation and suggest that the rapidly evolving E3 genes codify multifunctional proteins that target disparate host factors to modulate diverse host processes. Such economic and widespread targeting has been observed in intracellular targeting for a number of viral pathogens^1^,^2^,^33^, but has not been demonstrated for extracellular viral–host interactions or across viral species and types.

These results reveal conserved immunoregulatory strategies as well as a broad viral type-specific host factor targeting. In particular, we found that non-homologous E3 genes from different HAdV species target common and unique SLAM family receptors, which importantly, have been characterized with regards to inhibitory processes^27^,^34^,^35^,^36^. Similarly, interaction with the inhibitory receptors LILRB1 and LILRB2 was conserved in most HAdV species. A plausible explanation for this observation is that HAdVs have evolved decoy proteins capable of activating the inhibitory receptor LILRB1 as a mechanism to abrogate NK-mediated recognition of infected cells upon MHC-I downregulation triggered by the conserved E3/19K protein^37^. These data point towards important unappreciated roles for these receptors in adenovirus immunity. It will be of interest to elucidate whether overlapping patterns are found within other virus–host interactomes. We demonstrate that the LIL and SLAM receptor-interacting E3 proteins are capable of promoting receptor activation diminishing immune cell functions. LILRB1 and LILRB2 show partially overlapping yet distinct expression patterns^20^, whereas SLAM receptors are broadly distributed in immune cells and several members of the family are found co-expressed^17^,^18^, Whether these receptors play overlapping or unique functions remains poorly understood but the observation that particular viral proteins target several SLAMs, as well as either LILRB1 or LILRB2, suggest differential functions for each receptor. The landscape of host–pathogen interaction takes place in complex and evolving microenvironments within tissues, resulting in heterogeneous outcomes that depend on both the pathogen and host genetic factors^38^. Selective targeting of LILRB1 or LILRB2, similarly to specific or concerted modulation of SLAM receptors, may provide a selective advantage within certain microenvironments or unknown immunological contexts that could ultimately contribute to the differential pathological outcome characteristic of each HAdV species. Interestingly, an increased expression of LILRB1, LILRB2 or specific SLAM members has been associated with T cell dysfunction during a number of chronic viral infections^39^,^40^,^41^. Several HAdV species have been shown to persist in the infected individual, through mechanisms that are not understood^42^,^43^. It is tempting to speculate that the prominent targeting of inhibitory receptors observed for HAdV contributes to ameliorating T-cell responses over the course of the infection, especially during the late stages characterized by high levels of inhibitory receptors and hyporeactive immune cells. It will be important to study whether manipulation of the receptors described here plays a role in HAdV persistence in the organism, and more importantly, if similar tactics have been evolved by prominent chronic pathogens such as herpes viruses.

Interestingly, we identified binding of the HAdV-D47 E3/49K protein to the CD300A and CD300C paired receptors, receptor families composed by highly related inhibitory and activating members often co-expressed in cells^44^,^45^. To date, the only viral ligand known to target both activating and inhibitory members of a paired receptor family is m157, a protein encoded by murine cytomegalovirus (CMV) that binds both activating and inhibitory Ly49 receptors on NK cells, leading to host protection or susceptibility to disease, respectively^46^. Our observation suggests that similar to CMV, HAdV have evolved effectors to modulate unknown co-regulatory mechanisms involving certain paired-receptor systems for immunoevasion. It has been hypothesized that rapidly evolving pathogen-encoded proteins provide the selective pressure driving diversification of the paired receptor families, as well as other regulatory receptors such as CD45 (refs 46, 47). Consistent with this notion, our analysis revealed CD45 targeting as a highly conserved function, which interestingly, was limited to species D, suggesting a central role in the pathogenesis of this viral species. Moreover, we found that two viral types commonly associated with EKC, viral types 19a and 37, additionally interacted with the ISLR2 receptor. ISLR2 has been shown to play a relevant role in the modulation of processes such as axon growth and guidance^48^. Besides ISLR2, we identified PUNC, NELL2 and EPHA3 among others, as HAdV-D type-specific targets, factors known to have relevant roles in the nervous system^49^,^50^,^51^. Intriguingly, the nervous system has been identified as a common anatomical site of adenovirus persistence, predominantly for species D viruses^52^,^53^. Further studies may address whether targeting of these host factors in combination with CD45 synergistically influences the immune response and plays a role in species D-associated eye disease or the neurotropism of certain viral types.

Our study also revealed receptors, such as MPZL1, EPHA6 or PUNC, which have not been described as viral targets nor have been implicated in immune processes. Several of these receptors are expressed in both immune and non-immune cells, where they play known or predicted roles in cell communication, motility or adhesion. It is conceivable that some of the receptors we identify as targets for HAdVs play unrecognized functions in the immune response, such as the modulation of adhesion processes between immune cells or components of the extracellular matrix. Further study of these molecules may advance our understanding of the basic biology of the immune system and allow the design of novel strategies of therapeutic interest.

The E3 proteins represent a rapidly evolving component of the adenovirus genome that is likely evolving with the host immune system, driving the diversification of the viral family along with species-specific pathology and tropism. Our work provides relevant insights into potential viral perturbation of the immune system, results that may prove valuable for designing new or improved antiviral strategies. Future studies should address whether the receptors and pathways uncovered here play a role during viral infection in humans. Further characterization of the novel interactions identified here may also provide a rationale for engineering improved HAdV vectors, based on the incorporation or elimination of immunoregulatory functions encoded by particular E3 genes. The present work provides a reference for HAdV immunomodulation, and uncovers prominent immunoregulatory strategies that may be utilized by other human pathogens.

## Methods

### Cell work and viral strain

Human adenovirus 28 strain BP-5 was purchased from ATCC (reference VR-226). HEK293T, CHO and HeLa cells were cultured in DMEM high glucose medium supplemented with 10% FBS, glutamine and antibiotics. A549, K562, Jurkat, HEL, CML-T1 and primary cells were cultured in RPMI-1640 medium supplemented with 10% FBS, glutamine and antibiotics. Human umbilical vein endothelial cells (HUVEC) were purchased from Lonza, and cultured in EGM media supplemented following the manufacturer’s instructions. All the cell lines were obtained from the ATCC and cultured at 37 °C and 5% CO_2_ unless indicated. The blood samples were obtained from voluntary donors under Genentech blood donation program. In brief, PBMCs were isolated from heparinized blood by Ficoll (Lymphoprep) gradient centrifugation. Primary NK cells were isolated by negative selection using a NK cell isolation kit and following the manufacturer’s instructions (Miltenyi Biotec) and cultured overnight (O/N) in the presence of IL-2 (R&D Systems). Transient cell transfections were performed using Lipofectamine LTX and Plus reagent (Life Technologies) following the manufacturer’s instructions. Typically, the cells were transfected in six-well plates with 2 μg of the appropriate DNA. The HEK293T cells were incubated for 3 days after transfection, whereas the CHO cells were grown for 48 h before the assays.

### Cloning and protein purification

DNA sequences encoding each ECD were flanked by a 5′ ClaI restriction site and consensus kozak sequence (CCACC), and a 3′ AscI restriction site and ordered for synthesis by GeneArt (Life Technologies). The synthesized sequences were cloned into a pRK5 vector (Genentech) containing a carboxy (C)-terminal human IgG1 tag. HAdV-D53 E3/49K protein was subcloned in pRK5 vector containing a C-terminal EGFP-GPI tag to enable cell surface expression, as previously described^54^. Full-length versions for all receptors analysed were acquired from Invitrogen or ThermoScientific. The Fc fusion proteins were expressed and purified as previously described^55^. Briefly, the proteins were transiently expressed in CHO or HEK293S cells as 1-litre cultures and subsequently purified over HiTrap 5-ml MabSelect Sure columns (GE Healthcare). The protein was eluted with 50 mM citric acid, 150 mM NaCl, pH 3.0 and neutralized to pH 5 with 1 M arginine, 400 mM succinic acid pH 9.0. The protein concentration was determined by ultraviolet spectrometry at 280 nm using the theoretical extinction coefficient. Proteins showing less than 5% aggregation via size exclusion chromatography were dialysed into phosphate-buffered saline (PBS). The proteins with greater than 5% aggregation were purified further using size exclusion chromatography (HiLoad 16/600 Superdex 200 prep grade and Superdex 200 10/300 GL; GE Healthcare) and formulated as above. SDS–PAGE (polyacrylamide gel electrophoresis) was used to assess protein purity. Finally, protein identity and purity were evaluated by SDS–PAGE and mass spectrum analysis.

### Antibodies and protein reagents

The following antibodies were used for immunoprecipitation of the indicated receptors: SLAMF3 (HLy9.1, Novus Biologicals); SLAMF5 (Thermo Scientific); SLAMF6 (Novus Biologicals); LILRB1 (Santa Cruz Biotechnology). Antibodies were diluted 1:50 and incubated with the cell lysates at 4 °C O/N. For SLAM receptor detection by western blotting, goat polyclonal antibodies against SLAMF3 and SLAMF6, and a monoclonal antibody against SLAMF5, were purchased from R&D Systems. The membranes were incubated for 2 h at room temperature with the antibodies at the concentration indicated by the manufacturer, except in the case of the SLAMF3 blots which were incubated O/N. The following antibodies were used for cell stimulation: SLAMF3 (HLy9.1, Novus Biologicals), anti-Jurkat TCR (C305, Millipore) and anti-human IgG used for ephrin-A5 clustering (Jackson Immunoresearch) at the concentrations indicated in each assay. The following reagents were used to detect cell surface expression by flow cytometry: LILRB1 (BD Pharmingen); LILRB3 (Thermo Scientific); LILRB4 (US Biologicals); LILRB5, LILRA1, SLAMF3, SLAMF5 and SLAMF6 were acquired from R&D Systems; LILRA2 (GeneTex); LILRB2, LILRA5, LILRA6, ISLR2, MPZL1, CD300A, CD300C, SLAMF2, SLAMF4, SLAMF8 and SLAMF9 were purchased from Santa Cruz Biotechnology; CD45 (BD Pharmingen); LILRA4, PUNC, EPHA3 and EPHA6 antibodies were purchased from Novus Biologicals; SLAMF1 (Origene Technologies); SLAMF7 (Ad Serotec). Antibodies were typically used at a 1:50 or 1:100 dilution for flow cytometry analysis. The polyclonal anti-SLAMF3 antibody used for cell aggregation assays was from R&D Systems. The anti-phosphotyrosine antibody used for western blot detection was purchased from R&D Systems, anti-phospho-ERK1/2 and phospho-SHP-1 antibodies were from Cell Signalling and the anti-phospho-MPZL1 antibody was purchased from US Biological. Alexa 488-conjugated phalloidin was acquired from Life Technologies, and the Alexa 647-conjugated anti-phospho-tyrosine was purchased from BioLegend. All antibodies were used at the concentrations recommended by the manufacturer. The Hsp70 antibody used for western blot was purchased from Novus Biologicals and utilized at a 1:500 dilution. Isotype controls used for flow cytometry and cell stimulation were all purchased from BD Pharmingen.

The polyclonal antibody against HAdV-D28 E3/49K ECD was generated in house following the standard protocols. In brief, New Zealand white rabbits were immunized with 0.5 mg of antigen emulsified in Complete Freund’s Adjuvant for priming and subsequent immunizations, which were given at 2-week intervals. Following antibody production, serum was purified by E3/49K antigen-specific affinity chromatography.

The following recombinant purified ECD expressed as Fc-fusion proteins, except for IL-22 that was untagged, were purchased from R&D Systems: LILRB1, EPHA3, ephrin-A5, SLAMF4, SLAMF8 and SLAMF9. His-tagged SLAMF3 was purchased from R&D Systems. Fc-tagged SLAMF6 was from Sino Biologicals. Additional recombinant proteins used for validation purposes as well as the complete set of viral proteins studied, were purified in house as Fc-tagged recombinant proteins following the procedure indicated above; IL-11 was produced as a Flag-tagged recombinant protein and CD45RO ECD was purified as a His-tagged protein.

### Microarray processing and data analysis

Preparation of the human extracellular libraries, as well as the implementation of the protein microarray platform for extracellular protein–protein interaction discovery have been described in detail previously^5^,^6^. Labelling of viral Fc-tagged proteins and the formation of protein A microbead complexes was performed as described^5^. In brief, an immunoglobulin (IgG), used as an inert carrier, was labelled with a Cy5 monoreactive dye (GE Healthcare). To form the multivalent-microbead complexes, the Cy5-labelled IgG and the viral Fc–fusion protein were incubated with protein A microbeads (Miltenyi Biotec) and then the complexes were supplemented with 1 mg ml^−1^ soluble protein A (Sigma) immediately before the binding assay to block nonspecific binding to the protein microarray slides. Slides were loaded onto an automated a-Hyb hybridization station (Miltenyi) following the same protocol described previously^5^ and subsequently scanned with a GenePix 4000B scanner (Molecular Devices). GenePix Pro 6.0 software (Molecular Devices) was used for analysis. The data processing, analysis and scoring was performed as previously described with minor modifications^6^. The protein array analysis was done with a *z*-score based on the array distribution and scores were standardized with quantile normalization. Probes that bound to 5% or more of the baits, as determined by proportions test of a representative set of screens, were flagged as nonspecific binders (tallies of binding events from the representative set were counted if they scored 3.30 or higher). After eliminating nonspecific binders, probes with a score of 5.0 or higher were counted as hits. To control for slide variability, duplicate microarrays from separate spotting runs were analysed in all cases.

### SPR validation and binding affinity determination

Candidate hits were validated by SPR using a Biacore 3000 (GE Healthcare) or a Proteon XPR36 (Bio-Rad) instrument. Proteins were immobilized on CM5 sensor chips using the amino-coupling method. For Proteon measurements, GLC sensor chips were utilized. Analytes were run at the concentrations indicated in each case in HBS-P buffer (0.01 M Hepes, 0.15 M NaCl and 0.005% surfactant P20, pH 7.4) or PBS containing 0.05% Tween when the Proteon instrument was used. All Biacore sensorgrams were analysed with the Biaevaluation software 4.1. Bulk refractive index changes were removed by substrating the reference flow responses, and the average response of a blank injection was subtracted from all analyte sensorgrams to remove systematic artifacts. Proteon data were processed and analysed with the Proteon manager 3.1.0.6 software. Kinetic parameters were calculated using the Proteon XPR36 instrument and kinetic data were fit to a bivalent analyte model.

### Flow cytometry

The cells were stained with specific antibodies or isotype controls using standard flow cytometry procedures and processed on a FACSCanto II instrument (BD). In some cases, the automated plate harvester of the FACSCanto was used for uniformity of cell processing. Samples were analysed using Flow Jo 9.2 software (Tree Star). When leukocytic cell lines were analysed for protein binding or receptor expression, the cells were treated with an Fc receptor-blocking reagent (BioLegend). For binding analysis, HEK293T cells (typically 2 × 10^5^) were incubated with the viral proteins at the concentrations indicated during 30 min on ice, washed and fixed with 2% paraformaldehyde before analysis. Protein binding was assessed using PE- or APC-conjugated anti-human IgG-specific antibodies (Jackson ImmunoResearch).

### Immunoprecipitation and western blotting

In all the experiments, the cells were washed with cold PBS after stimulation, lysed with constant rotation during 30 min in lysis buffer (20 mM Tris buffer pH 7.4, 100 mM NaCl, 5% glycerol and 10% NP-40) containing tyrosine phosphatase and protease inhibitors (Sigma) and subsequently spun down at 18,000*g* during 10 min to remove nuclei. For LILRB1 immunoprecipitation experiments, the cells were lysed in buffer containing 1% Triton X-100. For immunoprecipitation, lysates were incubated O/N with the appropriate antibodies at the concentrations indicated above followed by incubation with protein G or protein A microbeads (Miltenyi) during 3 h. After incubation, the beads were washed with lysis buffer and immunocomplexes were eluted by boiling in SDS–PAGE sample buffer. All the procedures were performed at 4 °C. The samples were run in 4–20% Tris-glycine gels (Novex) and subsequently transferred to nitrocellulose membranes for western blot analysis. The blots were probed for proteins of interest using appropriate antibodies as indicated, followed by incubation with HRP-conjugated secondary antibodies (Jackson Immunoresearch). The membranes were developed using the ECL plus western blotting substrate (Thermo Scientific). For SLAMF3 stimulation, the cells were incubated for 5 min with the viral or control proteins (Genentech) at the molar concentrations indicated or with a combination of anti-TCR and anti-SLAMF3 antibodies used at 1 μg ml^−1^ and 5 μg ml^−1^, respectively. For SLAMF5 and SLAMF6 immunoprecipitation assays, HEL cells, which express high endogenous levels of both receptors (not shown), were stimulated with the proteins indicated (50 nM) for 5 min. For LILRB1 activation analysis, PBMCs were isolated as described, rested O/N and then stimulated with the proteins indicated for 5 min.

### E3/49K localization and binding of hits to infected cells

The A549 cells were infected with HAdV-D28 at a multiplicity of infection (MOI) of 0.5. The expression of the E3/49K protein at the surface of infected cells was analysed at the indicated time points post infection by flow cytometry using the α-HAdV-D28 E3/49K polyclonal antibody generated in house. In brief, the infected cells were collected and incubated with the α-E3/49K antibody for 30 min at 4 °C. After washing, the cells were fixed with 4% paraformaldehyde (PFA) and stained with APC-conjugated α-rabbit IgG antibody (Jackson ImmunoResearch). For immunofluorescence detection, the cells were seeded on eight-well chambered coverslips (Lab-Tek) and infected at low MOI for 32 h. The expression of E3/49K protein at the surface was detected using the α-E3/49K polyclonal antibody followed by incubation with an Alexa 488-conjugated α-rabbit IgG (Invitrogen). The images were acquired on a Zeiss Axiovert 200 M inverted microscope and processed using Fiji software. To analyse E3/49K secretion from infected cells, supernatants were harvested at the indicated times post infection and spun down at 1,000*g*. The samples were diluted in SDS–PAGE reducing loading buffer and boiled for western blot analysis. The membranes were probed with the HAdV-D28 E3/49K polyclonal antibody followed by peroxidase-conjugated α-rabbit IgG (Jackson ImmunoResearch).

For the analysis of receptor binding to cells, the HAdV-D28-infected cells (32 h post infection) were incubated with 1 μg of CD45-His, LILRB1-Fc or TREM1-Fc purified recombinant ECDs, washed and fixed for 15 min in 4% PFA. Protein binding was detected with APC-labelled anti-human IgG (Jackson ImmunoResearch), followed by analysis on a BD Canto II flow cytometer (BD Biosciences).

### Knockdown of SLAMF receptors in Jurkat cells

SLAMF5 was silenced in Jurkat T cells using MISSION lentiviral transduction particles (Sigma), using the TRCN0000057476 clone, Briefly, 2 × 10^5^ cells were infected at different MOI by spinoculation, as previously described^56^ and subsequently cultured for 3 days. Afterwards, the growth media was replaced with fresh RPMI media containing puromycin at 1 μg ml^−1^. The cells were maintained in puromycin until the non-transduced control cells died as determined by Trypan Blue staining. Resistant colonies were further expanded and analysed for receptor expression by flow cytometry previous to binding and T-cell activation assays in the presence of the viral proteins.

### Analysis of MAPK ERK activation

To analyse ERK1/2 phosphorylation, Jurkat cells were starved and then pre-incubated with the indicated proteins (50 nM) for 15 min and subsequently stimulated with the anti-TCR C305 antibody (Millipore; 0.65 μg ml^−1^) for 15 min, similarly to previously described^57^. The cell lysates were processed as described in the ‘Immunoprecipitation and western blotting’ section.

### Gene ontology analysis

Host factors found to interact with the HAdV E3 proteins were subjected to Gene Ontology analysis using the GOstats Bioconductor package^58^. Host genes were split by the originating species of the E3 protein bound. Each gene list was then queried for enriched GO biological process terms, using a hypergeometric test. GO terms were selected at an unadjusted *P* value of 0.01, with at least two host proteins in a GO category for it to be included in the analysis. Because we wanted to contrast the different gene lists, we used a non-conditional GO analysis (that is, not considering the topology of the GO tree). This leads to highly redundant selection of GO categories, so the final list was pruned to just include the most specific terms that were enriched. The GO terms were renamed for simplicity. The new terms and associated numerical *P* values for each HAdV species are shown in Supplementary Table 2.

### Disease association analysis

Host factors targeted by the HAdV E3 proteins were mapped to gene–disease associations annotated in the DisGeNET database^22^ (v.1). Curated, predicted and literature databases were searched for host gene–disease association, selecting both therapeutic and biomarker association types. The association of each host protein with disease was further checked for accuracy using the literature references found in the DisGeNET database. Disease terms from the DisGeNET database were manually collapsed to create curated terms for the network, as shown in the Supplementary Information. Cytoscape was used to generate the disease association pie charts.

### Cell adhesion assays

For adhesion assays with HeLa cells, 96-well flat-bottom plates were coated with FN or collagen (Invitrogen) at the concentrations indicated during 2 h at 37 °C or O/N at 4 °C, and then incubated with 1% bovine serum albumin (BSA) for 30 min before the assay or washed with PBS, respectively. The cells were serum starved and then incubated (2 × 10^5^ per well) with the indicated viral proteins (200 nM) for 15 min in DMEM media containing 0.1% BSA and then seeded on FN- or collagen-coated plates for 50 min at 37 °C. The unbound cells were washed away and remaining attached cells were fixed with 4% PFA and subsequently stained with 0.5% crystal violet. After extensive washing, the adhered cells were quantified by measuring the optical density at 560 nM in a microtiter plate reader.

For HUVEC-leukocyte adhesion assays, the HUVEC cells were grown on 96-well black fluorescent plates to ≈80% confluency. The cell adhesion was analysed using the endothelial cell adhesion kit (Millipore), following the manufacturer’s instructions. In brief, the endothelial cell monolayers were starved for 1 h and then activated with TNFalpha for 6 h at 37 °C. Afterwards, Calcein AM-labelled CML-T1 T cells were incubated with the HUVEC cells for 30 min at 37 °C. The CML-T1 cells were incubated with the indicated stimuli for 30 min before the assay. The α-ICAM antibody provided with the kit was used at 25 μg ml^−1^, whereas E3 or hIgG control proteins were added at 100 nM concentration. After several mild washing steps, the amount of CML-T1 cells bound to the endothelial cells was quantified using a fluorescence plate reader (485/530 filter sets).

### Actin cytoskeleton and EPHA3-ephrin-A5 assays

HAdV-D43 E3/49K protein binding to cells was analysed by immunofluorescence. In brief, the HEK293T cells cultured on poly-D-lysine-coated plates were transfected with the EPHA3 receptor for 24 h, and afterwards incubated with the E3/49K proteins (100 nM) for 30 min on ice, washed and then the protein bound to the surface was detected using an Alexa 555-conjugated α-human Ig antibody (Life Technologies). The cells were fixed with 2% PFA and the images were acquired using a Leica SP inverted confocal. To analyse the changes in the actin cytoskeleton, the HEK293T cells transiently transfected with the EPHA3 receptor were incubated with the indicated proteins (100 nM) for 20 min at 37 °C or mock-treated, followed by stimulation with ephrin-A5 for 5 min when indicated. The cells were incubated on FN-coated plates (at 10 μg ml^−1^) for 60 min at 37 °C, washed, fixed with 4% PFA, permeabilized using Tritron X-100 and blocked with PBS containing 0.2% BSA. Afterwards, the actin cytoskeleton was stained with Alexa 488-conjugated Phalloidin (Invitrogen) and tyrosine phosphorylation was analysed using an Alexa 647-conjugated α-phosphotyrosine antibody (PY20, BioLegend). The images were acquired using a Leica SP inverted confocal at × 63 magnification and represent maximal projections of Z-stacks to allow visualization of the body cell height from bottom to top. The images were processed using Fiji software. For the analysis of cell adhesion, the cells were stimulated following the same procedure and then allowed to attach to FN-coated plates for 60 min at 37 °C. Afterwards cell adhesion was quantified as described for HeLa cells. In all the cases, Fc-tagged ephrin-A5 (R&D Systems) was pre-clustered with an anti-human IgG antibody (Jackson Immunoresearch) at a 1:10 molar ratio for 20 min at 4 °C.

### Cell aggregation assays

Cell clustering assays were performed as previously described, with minor modifications^59^. The CHO cells were labelled with Vybrant DiD or CFSE dyes (Life Technologies) following the manufacturer’s instructions. The CFSE-labelled cells (2 × 10^5^) were pre-incubated with the indicated proteins (500 nM) or antibodies (5 μg ml^−1^) for 30 min at room temperature before co-incubation with the Vybrant-DiD-stained cells (2 × 10^5^) in PBS containing 1% BSA. The cells were gently spun down, incubated at 37 °C for 30 min in 5-ml polystyrene tubes and fixed with PFA for further analysis. Incubation with the wt non-transfected cells was used as a control for SLAMF3-mediated cell contacts. The images were acquired using a Leica SP5 inverted confocal system using × 20 objective. The stack images were processed using the Fiji 1.48b.

### Analysis of MPLZ1 activation

HeLa cells were starved in FBS-free DMEM media, detached with trypsin-free solution (Life Technologies) and incubated with the indicated viral proteins for 15 min at 37 °C. The samples were subjected to SDS–PAGE analysis using Bis-Tris gels (Novex) and subsequently transferred to PVDF membranes and analysed by western blotting using a specific anti-phospho-MPZL1 antibody (Cell Signaling). The membranes were reblotted using an anti-beta-actin antibody (Cell Signaling Technology) to control for equivalent protein loading.

### NK cell-mediated cell lysis

Primary NK cell-mediated killing of the susceptible K562 cell line was quantified using lactate dehydrogenase (LDH) release as readout for target cell lysis (LDH cytotoxicity assay kit, Thermo Scientific). The NK cells were incubated with the indicated proteins (100 nM) for 20 min and then added to K562 cells at varying effector:target ratios for 3 h before reading.

### Statistical analysis

Statistical analyses of data were performed with the program GraphPad Prism. Comparisons were performed using analysis of variance with Bonferroni *post hoc* test, or where appropriate with Students *t*-test, as indicated in each case.

## Additional information

**Accession codes:** The protein microarray data have been deposited in the GEO database under accession code GSE78518.

**How to cite this article:** Martinez-Martin, N. *et al*. The extracellular interactome of the human adenovirus family reveals diverse strategies for immunomodulation. *Nat. Commun.* 7:11473 doi: 10.1038/ncomms11473 (2016).

## Supplementary Material

Supplementary Information Supplementary Figures 1-8 and Supplementary Tables 1-3.

Supplementary Data 1 Contains the raw data after statistical analysis for hit calling following the procedures described in the Methods section.

## Figures and Tables

**Table 1:** Summary of the interactions identified and validated for all viral proteins analysed.

**Virus type/gene**	**Hit identification/technology**	**Hit validation/technology**	**Functional effect**
HAdV-A18/CR1α	SLAMF5/PM, SLAMF6/PM, SLAMF7/PM, IL-22/PM	SLAMF5/SPR, FC; SLAMF6/SPR, FC; SLAMF7/SPR, FC; IL-22/SPR	Increases SLAMF5 phosphorylation and inhibits T-cell activation in a SLAMF5-dependent manner
HAdV-B55/CR1α	NH	—	—
HAdV-D28/CR1α	LILRB2/FC	LILRB2/SPR	—
HAdV-E4/CR1α	NH	—	—
HAdV-F41/CR1α	NH	—	—
HAdV-G52/CR1α	NH	—	—
HAdV-A18/CR1β	SLAMF4/FC	SLAMF4/SPR	—
HAdV-B55/CR1β	SLAMF6/PM	SLAMF6/SPR, FC	Increases SLAMF6 phosphorylation and inhibits T-cell activation
HAdV-E4/CR1β	LILRB1/FC	LILRB1/SPR	Increases LILRB1 phosphorylation and inhibits NK cytotoxicity
HAdV-F41/CR1β	SLAMF4/PM; TIM3/PM; LAIR-1/PM; LILRB2/FC	SLAMF4/SPR, FC; TIM3/SPR; LAIR-1/SPR; LILRB2/SPR, FC	—
HAdV-G52/CR1β	NH	—	—
HAdV-B1/CR1γ	SLAMF1/SPR; SLAMF3/SPR; SLAMF4/SPR; SLAMF7/SPR; SLAMF8/SPR	—	—
HAdV-D30/CR1γ	NH	—	—
HAdV-E4/CR1δ	NH	—	—
HAdV-D9/CR1β	CD45/PM	CD45/SPR, FC	—
HAdV-D19a/CR1β	CD45/PM; ISLR2/PM	CD45/SPR, FC; ISLR2/SPR, FC	—
HAdV-D23/CR1β	CD45/PM	CD45/SPR, FC	—
HAdV-D24/CR1β	CD45/PM; LILRB2/FC	CD45/SPR, FC; LILRB2/SPR	—
HAdV-D28/CR1β	CD45/PM; PUNC/PM; CST3/PM; NELL2/PM; LILRB1/FC	CD45/SPR, FC; PUNC/SPR, FC; CST3/SPR; NELL2/SPR; LILRB1/SPR	—
HAdV-D30/CR1β	CD45/PM; SLAMF3/PM; MPZL1/PM; IL-11/PM; DEFA3/PM; LILRB1/FC	CD45/SPR, FC; SLAMF3/SPR, FC; MPZL1/SPR, FC; IL-11/SPR; DEFA3/SPR; LILRB1/SPR	Increases SLAMF3 phosphorylation. Impairs SLAMF3-mediated cell adhesion. Increases MPZL1 activation and inhibits cell adhesion. Inhibits T-cell activation
HAdV-D37/CR1β	CD45/PM; ISLR2/PM; LILRB2/FC	CD45/SPR, FC; ISLR2/SPR, FC; LILRB2/SPR	—
HAdV-D39/CR1β	CD45/PM, LILRB1/PM	CD45/SPR, FC; LILRB1/SPR, FC	—
HAdV-D43/CR1β	CD45/PM; EPHA3/PM; EPHA6/PM; LILRB1/FC	CD45/SPR, FC; EPHA3/SPR, IF; EPHA6/SPR; LILRB1/SPR	Modulates EPHA3/ephrin-A5 effect on cell adhesion
HAdV-D47/CR1β	CD45/PM; CD300A/PM; CD300C/PM	CD45/SPR, FC; CD300A/SPR, FC; CD300C/SPR, FC	—
HAdV-D51/CR1β	CD45/PM, LILRB1/PM	CD45/SPR, FC; LILRB1/SPR, FC	—
HAdV-D53/CR1β	CD45/PM	CD45/SPR, FC	Inhibits NK cytotoxicity and T-cell activation
HAdV-D58/CR1β	CD45/PM	CD45/SPR, FC	—

FC, flow cytometry; HAdV, Human adenovirus; IF, immunofluorescence; NH, no hits identified; PM, protein microarray; SPR, surface plasmon resonance.A total of 27 HAdV E3 proteins encoded by six viral species were included in this study. 51 novel host interacting partners were identified for 20 HAdV proteins corresponding to five viral species. The technology utilized for hit identification and further experimental validation are indicated. The functional effect observed for the viral proteins studied is also summarized.
