# Corrigendum: The extracellular interactome of the human adenovirus family reveals diverse strategies for immunomodulation

**DOI:** 10.1038/ncomms16150

**Published:** 2017-08-21

**Authors:** Nadia Martinez-Martin, Sree R. Ramani, Jason A. Hackney, Irene Tom, Bernd J. Wranik, Michelle Chan, Johnny Wu, Maciej T. Paluch, Kentaro Takeda, Philip E. Hass, Hilary Clark, Lino C. Gonzalez

Nature Communications 7: Article number: 11473 ; DOI: 10.1038/ncomms11473 (2016); Published: 05 05 2016; Updated: 08 21 2017

In Supplementary Fig. 3 of this Article, the sequence given for CR1B_HAdV-D19a in the alignment shown is incorrect, and should have been identical to that listed for CR1B_HAdV-D37. A correct version of Supplementary Fig. 3 appears below as Figure 1, and a table of GenBank accession numbers and amino-acid boundaries used for cloning the viral proteins in this study appears below as Table 1.

The gene sequences utilized for production of the HAdV-D19a and HAdV-D37 CR1β recombinant proteins were correct. However, despite having identical sequences, a discrepancy was found for the binding partners identified for these two proteins in the subsequent interactome analysis: HAdV-D37 interacted with CD45, ISLR2 and LILRB2 receptors, whereas HAdV-D19a CR1β protein bound to CD45 and ISLR2. The reasons for these differences have not yet been conclusively determined; however, it should also have been noted that proteins D19 CR1b, D23 CR1b, D39 CR1b, D43 CR1b, D47 CR1b and D51 CR1b were expressed in CHO cells, whereas the remaining proteins, including D37 CR1b, were expressed in HEK293 cells. It is therefore possible that the use of different cell types underlies the differences in binding data. The specific proteins that were expressed in CHO cells should have been listed in the Methods section of the paper, where the cell types used were stated.

## Figures and Tables

**Figure 1:**
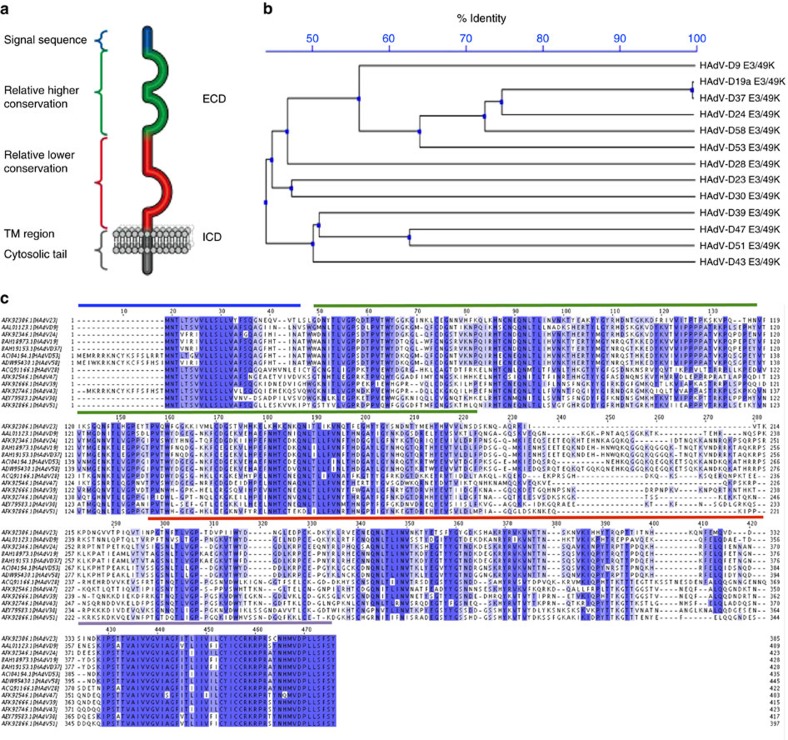


**Table 1:**
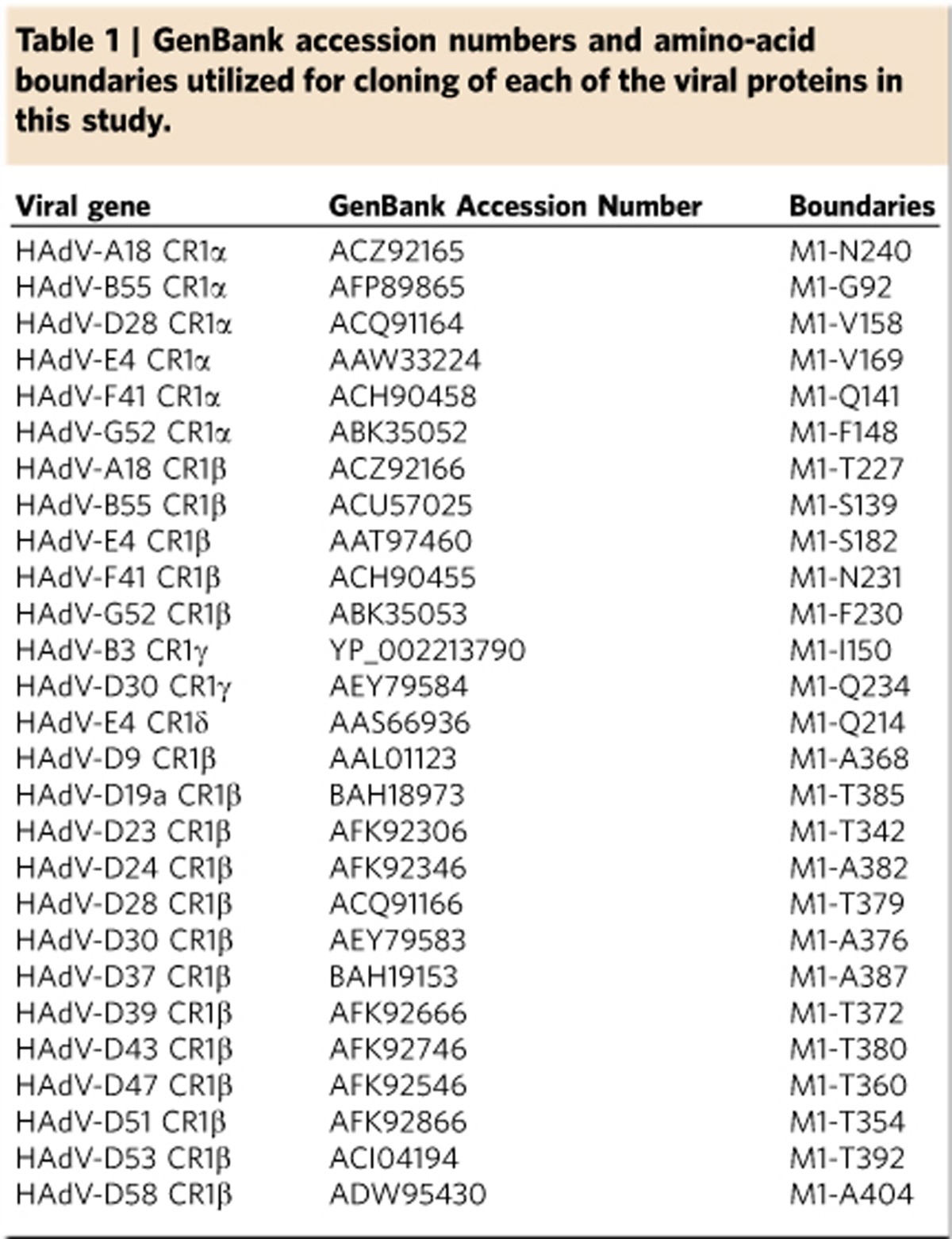
GenBank accession numbers and amino-acid boundaries utilized for cloning of each of the viral proteins in this study.

