# Supplementary material for: The extracellular interactome of the human adenovirus family reveals diverse strategies for immunomodulation
Source: Nat Commun. 2016 May 5;7:11473. doi: 10.1038/ncomms11473 (PMC4858740; doi:10.1038/ncomms11473)
Supplement: Supplementary Information — Supplementary Figures 1-8 and Supplementary Tables 1-3. [file ncomms11473-s1.pdf]

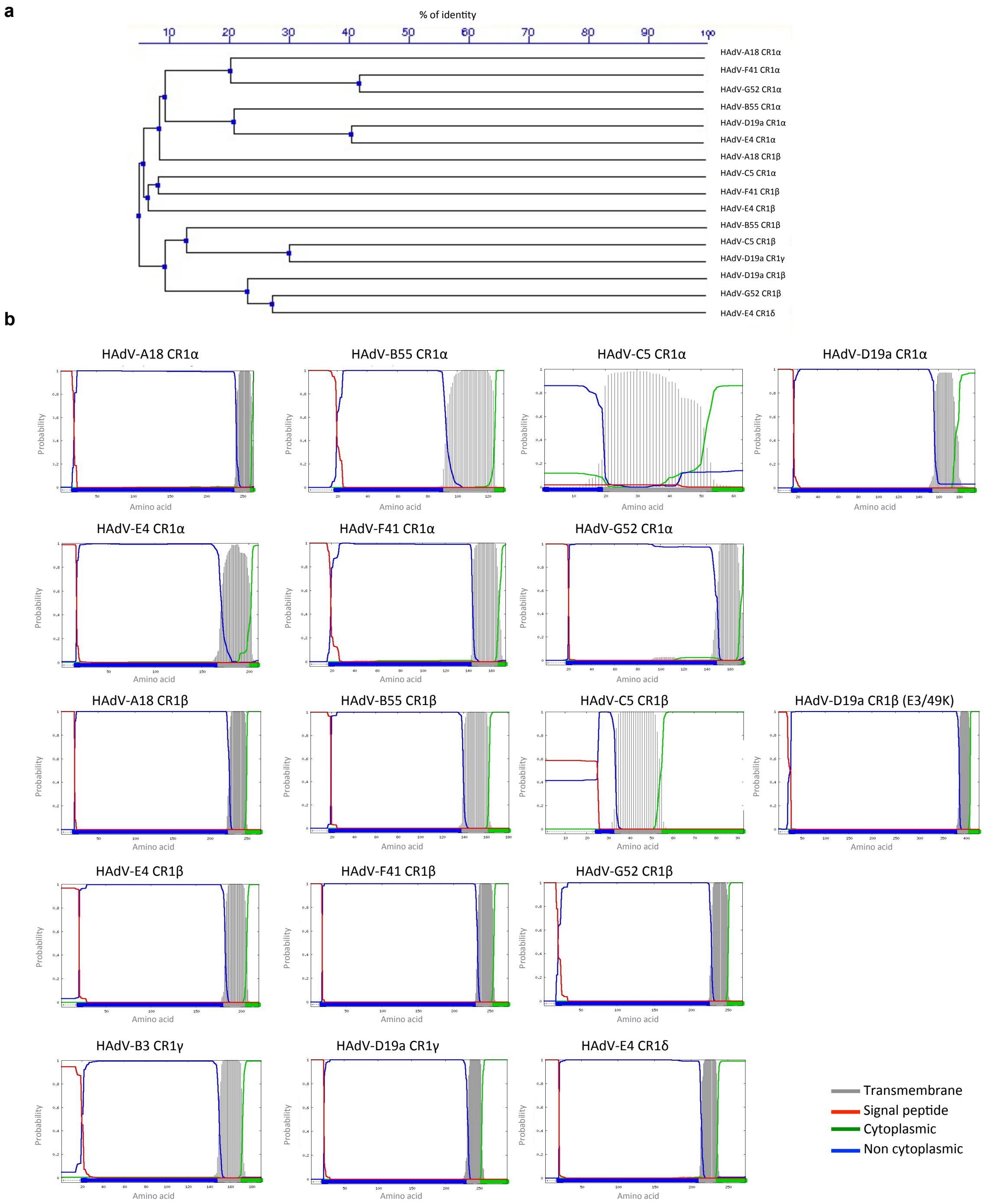


**Supplementary Figure 1 (associated to Figure 1). The highly divergent HAdV E3 genes encode for predicted extracellular proteins.** (**a**) UPGMA tree depicting the percentage of sequence identity shared by the HAdV E3 proteins analyzed in this study. (**b**) *In silico* prediction of transmembrane regions and signal peptides in the proteins encoded by the HAdV CR1α, CR1β, CR1γ and CR1δ genes. Phobius server was used to generate the plots shown.

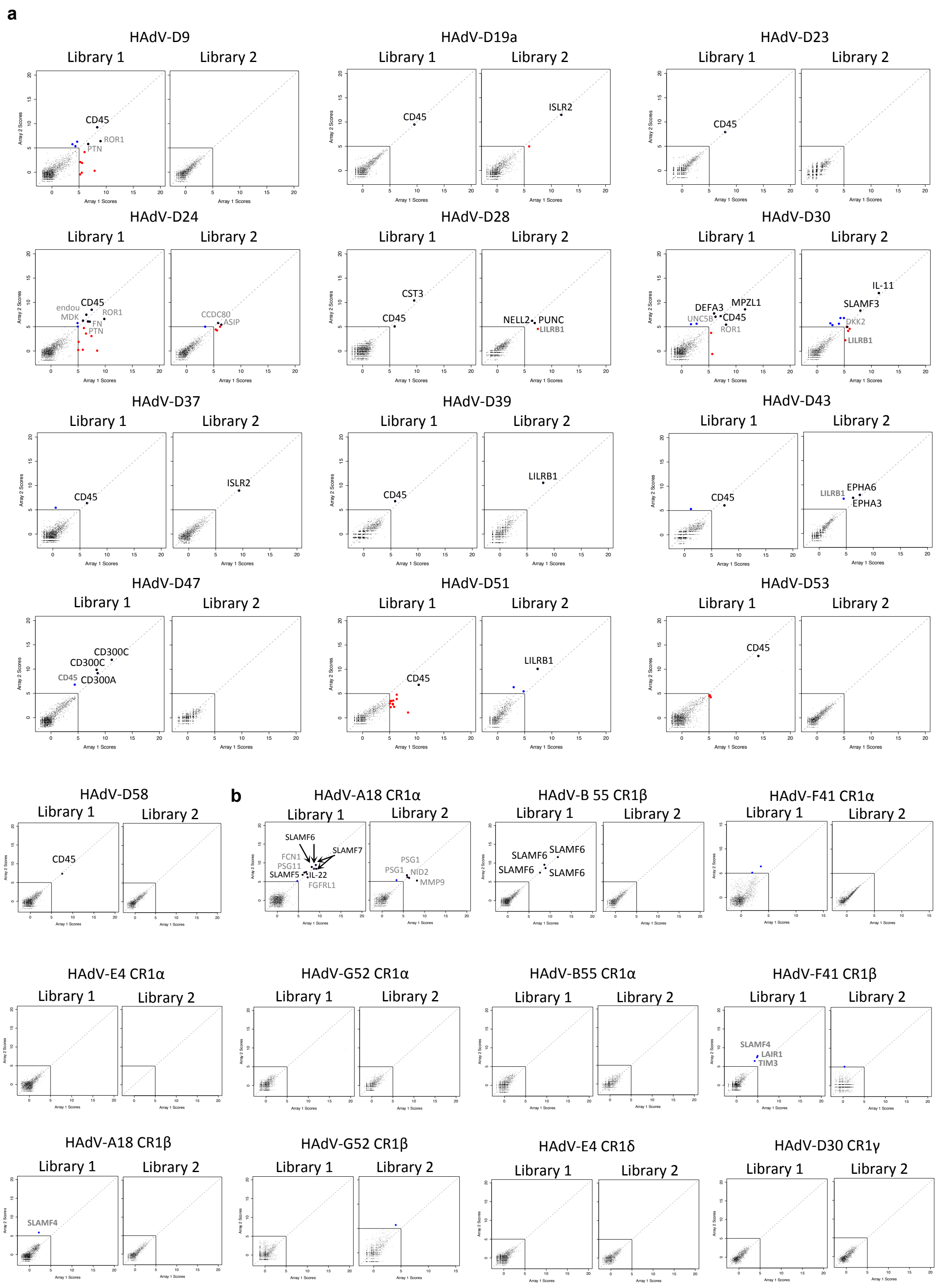


**Supplementary Figure 2 (associated to Figure 2). HAdV variability imparts conserved and type-specific targeting of host receptors.** (**a**) Intersection plots obtained for the HAdV species D E3/49K proteins selected for this study. Red and blue dots represent hits called on only a single array replicate, and black dots labeled in grey color indicate hits that were not selected for further

validation. (**b**) Intersection plots obtained for the rest of E3 proteins studied. Data were represented as in (a). The HAdV-D CR1α, HAdV-E CR1β and HAdV-B CR1γ proteins were also screened; however, the results obtained for these proteins could not be analyzed due to strong non-specific binding to the microarray slides (not shown).

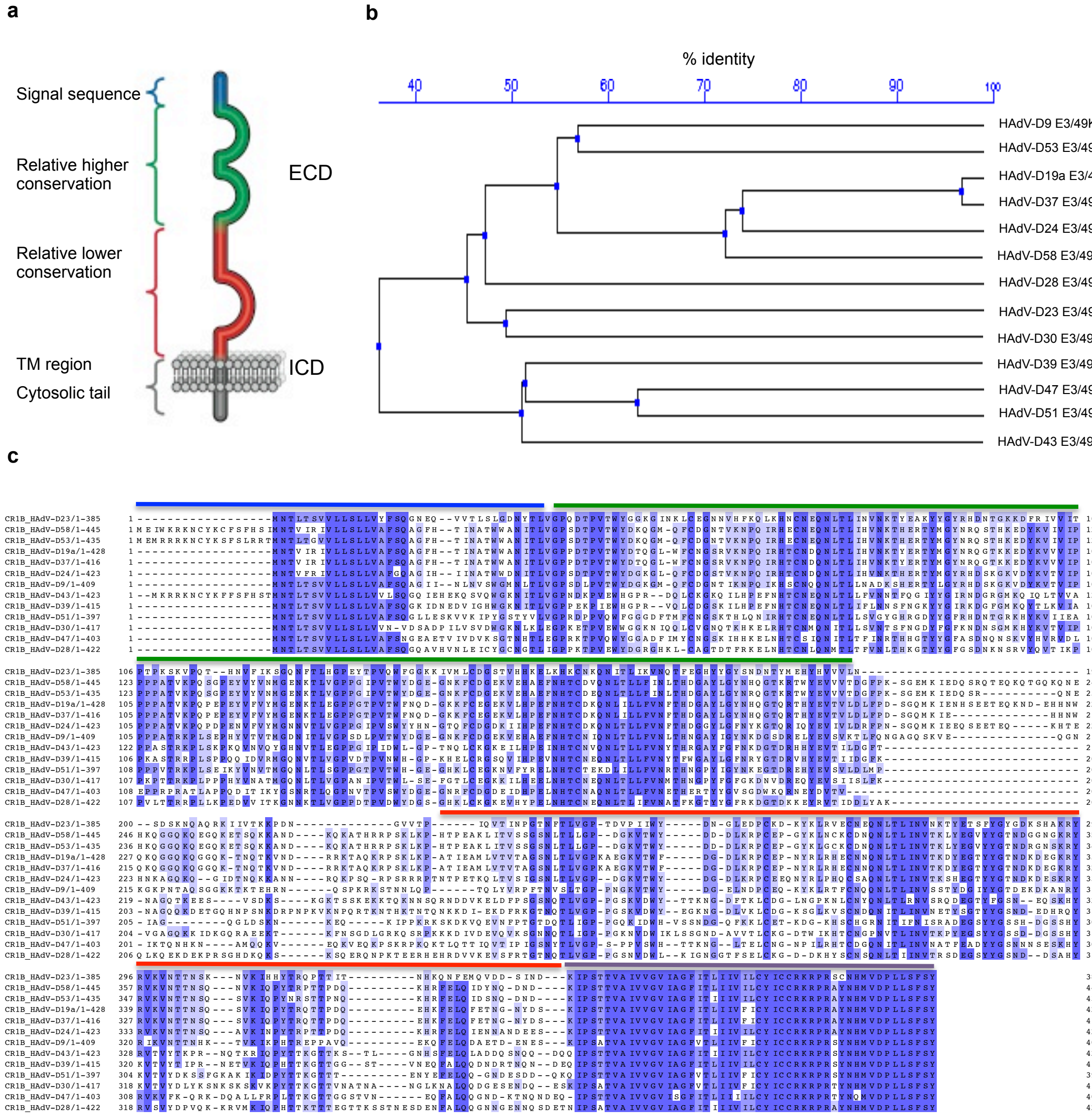


**Supplementary Figure 3 (associated to Figure 2). Sequence diversity of the HAdV-D E3/49K proteins studied.** (**a**) Schematic showing the E3/49K protein predicted structure, containing 3 predicted Ig-like domains followed by a transmembrane region and a short cytoplasmatic domain. (**b**) UPGMA tree showing the percentage of sequence identity shared by the 13 E3/49K proteins analyzed in this study. (**c**) Sequence alignment of the 13 E3/49K proteins studied. Blue bar indicates the position of the predicted signal peptide. Green bar shows the boundaries for Ig-like domains 1 and 2, which show a higher relative conservation within viral types. The region highlighted with a red bar represents the Ig-like domain 3, which shows the highest sequence variability among the E3/49K proteins. Purple bar indicates the transmembrane and the highly conserved intracellular regions. Abbreviations: ECD, extracellular domain; ICD, intracellular domain; TM, transmembrane region.

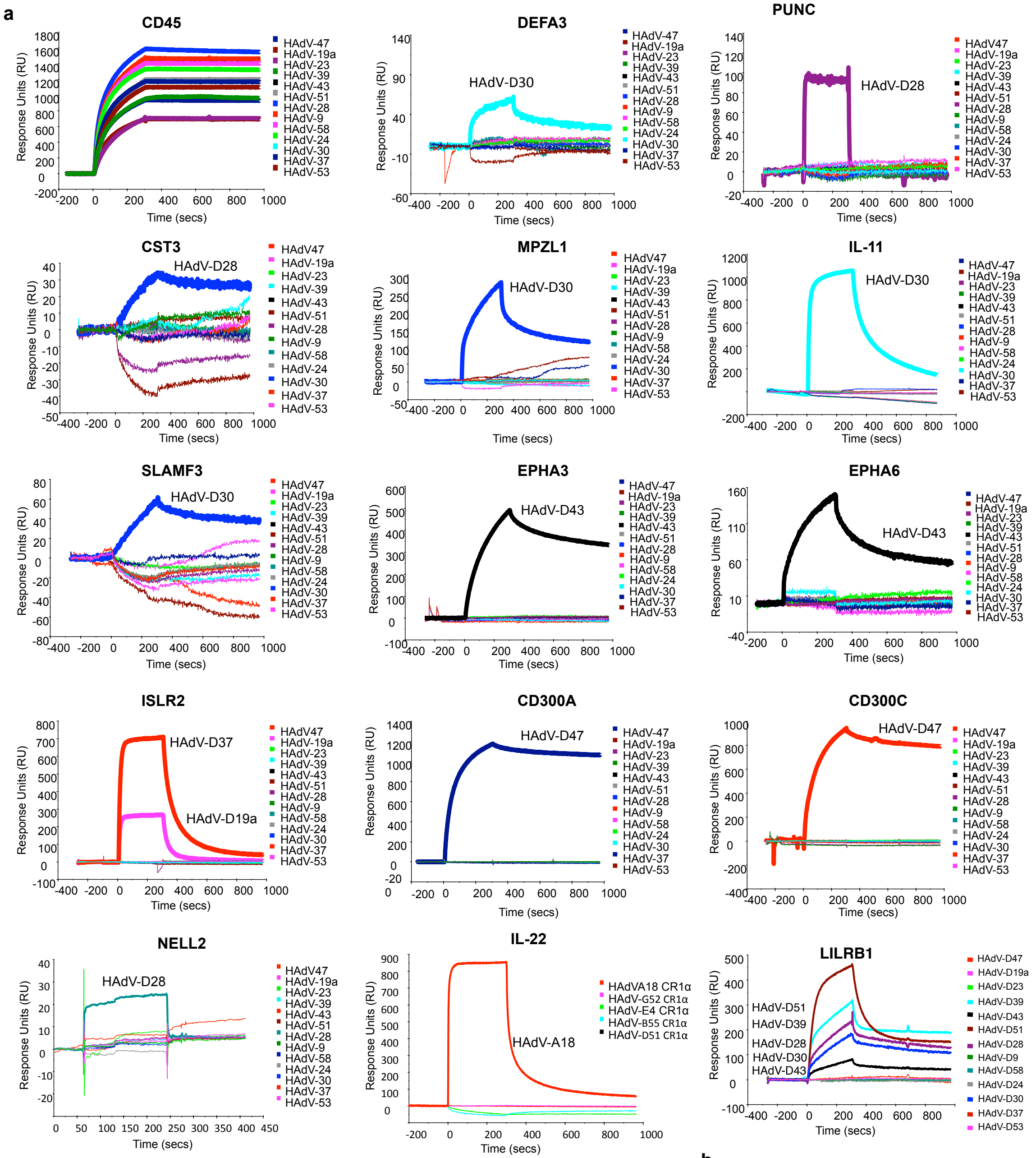


b

| E3/49K protein | $K_D$ (nM) ($\chi^2$) | Rmax |
|---|---|---|
| HAdV-D9 | 0.08 (46) | 1347 |
| HAdV-D19a | 0.06 (6.7) | 485 |
| HAdV-D23 | 0.18 (5) | 348 |
| HAdV-D24 | 0.08 (149) | 1795 |
| HAdV-D28 | 0.33 (9) | 745 |
| HAdV-D30 | 0.19 (12) | 554 |
| HAdV-D37 | 0.11 (50) | 1666 |
| HAdV-D39 | 0.39 (6) | 275 |
| HAdV-D43 | 0.38 (36) | 456 |
| HAdV-D47 | 0.01 (16) | 248 |
| HAdV-D51 | 0.06 (38) | 554 |
| HAdV-D53 | 0.21 (16) | 485 |
| HAdV-D58 | 0.38 (54) | 1473 |

**Supplementary Figure 4 (associated to Figure 2). Surface plasmon resonance validation of the E3 protein-host interactions identified and E3/49K CD45 binding affinity.** (**a**) All E3 proteins were immobilized on sensor chips and the ECD of the indicated human proteins,

expressed as purified recombinant proteins, were run as analytes. IL-11, IL-22, LILRB1 and LILRB2 were immobilized on sensor chips and the E3 proteins were run as analytes. Analyte concentrations used in each case are as follows: CD45, 25 nM; Defensin3A, 475 nM; PUNC, 55 nM; CST3, 61 nM; NELL2, 200 nM; IL-11, nM; SLAMF3, 100 nM; EPHA3, 59 nM; EPHA6, 59 nM; ISLR2, 58 nM; CD300A, 113 nM; CD300C, 113 nM; MPZL1, 122 nM; TIM3, 500 nM; SLAMF4, 100 nM; LAIR1, 500nM. IL-11 (16-60 nM, HAdV-D30 E3/49K was run at 18 nM; IL-22 (the indicated viral proteins were injected at 500 nM); LILRB1 and LILRB2 (E3 proteins injected at 200 nM). Similar RU (4000-6000) were immobilized in all cases. Positive binders have been labeled for the ease of visualization. Assays shown are representative of two independent assays. (**b**) Affinity constants for the interaction between the E3/49K proteins and recombinant purified CD45RO ECD. Binding affinities are shown as $K_D$ (nM) values. Rmax and $\chi^2$ values indicate the goodness of the experimental fitting.

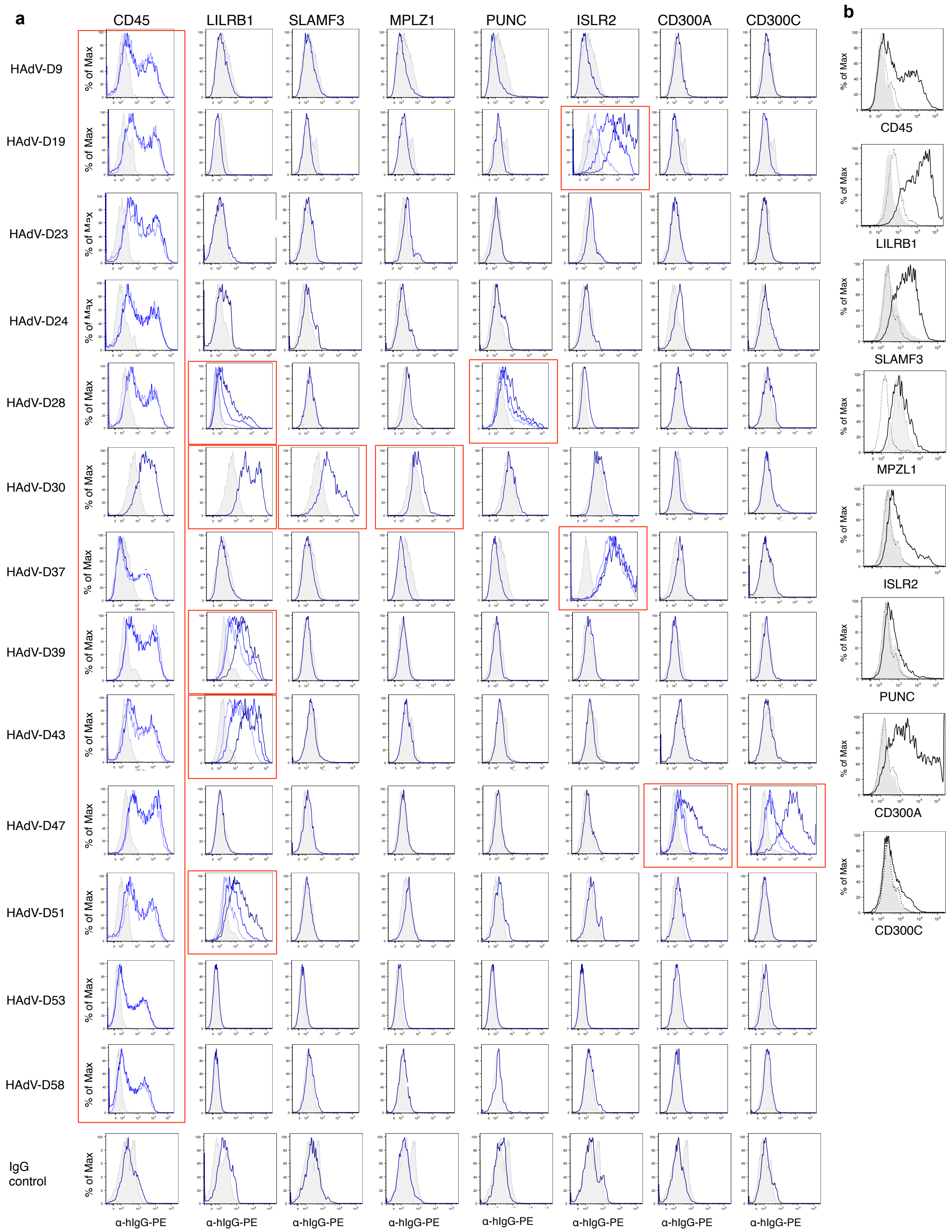


**Supplementary Figure 5 (associated to Figure 2). The E3/49K proteins specifically target the cell surface receptors identified.** (**a**) HEK293T cells were transfected with each of the receptors identified as hits and binding of soluble E3/49K proteins was analyzed by flow cytometry. Grey filled histograms represent binding of the E3/49K protein to wt cells (100 nM). Light, navy and dark blue histograms represent binding to the hit-transfected cells, at 1, 10 and

100 nM concentration, respectively. Due to binding of the HAdV-D30 E3/49K protein to non-transfected cells and for the ease of visualization, binding at one concentration is represented (100 nM). Red boxes indicate the interactions identified by the protein microarray screening. Histograms are representative of three independent assays. (**b**) Expression of each receptor on the surface of HEK293T cells used in a representative assay. Gray histograms show the expression of each receptor at the surface of non-transfected cells and black histograms indicate the surface expression in receptor-transfected cells, detected using specific antibodies. Dotted histogram represents isotype control binding.

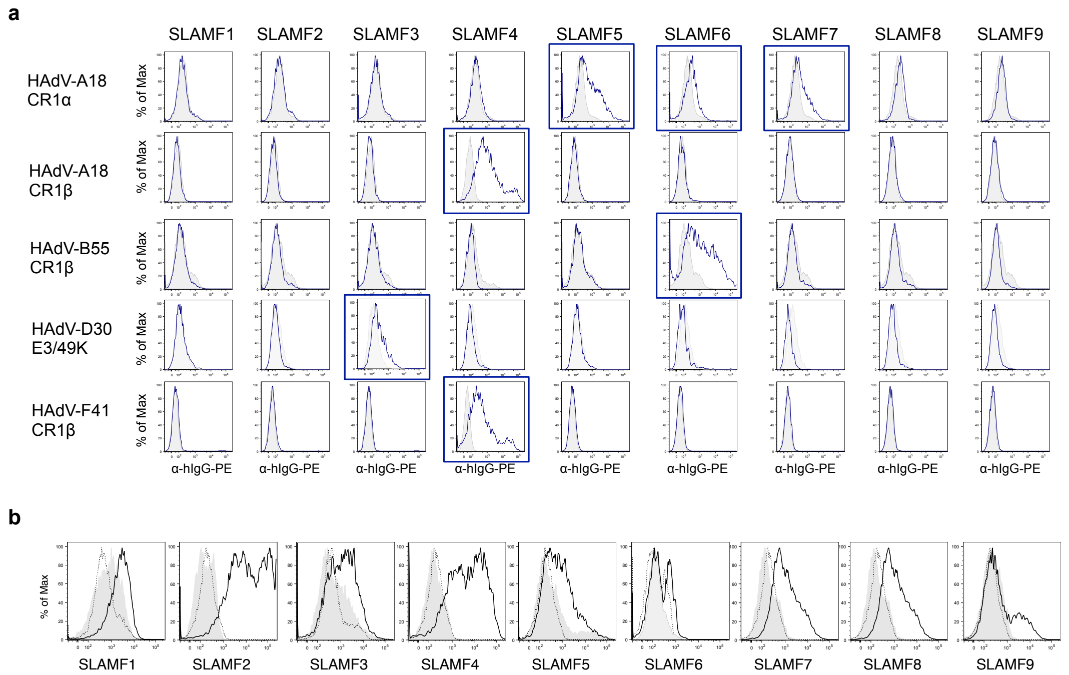


**Supplementary Figure 6 (associated to Figure 5).** (**a**) Binding of the E3 proteins to HEK293T cells expressing SLAM receptors. Grey and blue histograms represent binding to wt and SLAMF-transfected cells (200 nM), respectively. Blue boxes indicate the interactions identified. All E3 proteins were screened for binding to the SLAM receptor family; the HAdV-B CR1γ protein strongly interacted with wt cells precluding further analysis (not shown). (**b**) Expression of each SLAM receptor on the surface of HEK293T cells used in a representative assay. Gray histograms show the expression of each receptor at the surface of non-transfected cells and black histograms indicate the surface expression in receptor-transfected cells, detected using specific antibodies. Dotted histogram represents isotype control binding.

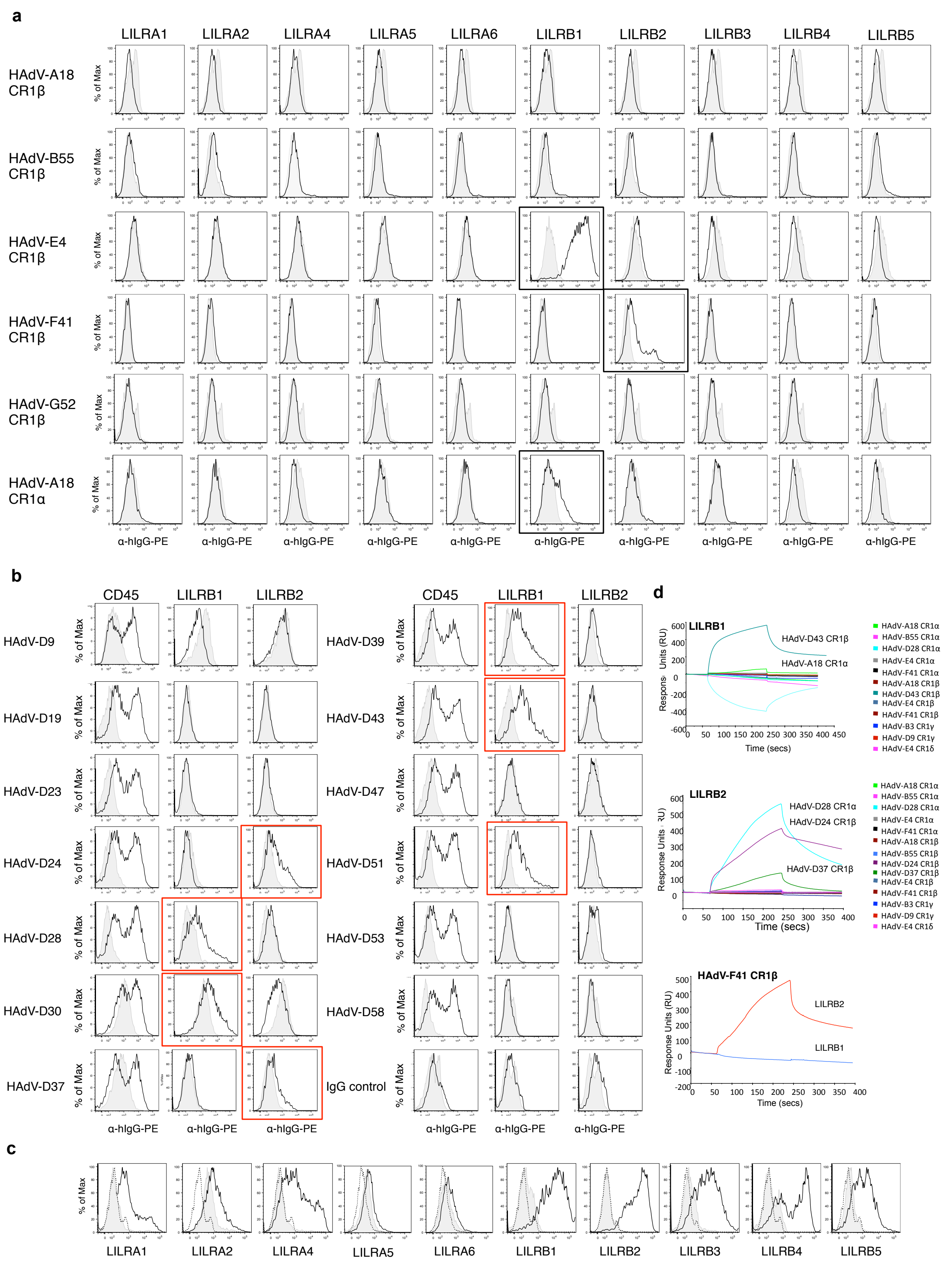


**Supplementary Figure 7 (associated to Figure 5). Interaction of HAdV E3 proteins with the LILR family.** (**a**) HEK293T cells were transfected with 10 LIL receptors and the binding of the indicated E3 proteins (200 nM) was analyzed by flow cytometry using an antibody directed against the Fc tag. The HAdV-E CR1β protein strongly interacted with surface LILRB1. HAdV-

F41 CR1β bound to LILRB2 whereas the HAdV-A18 CR1α targeted LILRB1. (**b**) Flow cytometry analysis of E3/49K binding (100 nM) to HEK293T cells transfected with LILRB1 or LILRB2. Gray and black histograms represent viral protein binding to wt and LILR-transfected cells, respectively. Red boxes indicate the interactions identified. Binding to the E3/49K proteins to all LIL receptors was analyzed by flow cytometry (not shown). (**c**) Histograms showing the expression of the LILR members at the surface of the cells used for the analysis. Gray histograms show the expression of each receptor at the surface of non-transfected cells. Receptor expression at the surface of transfected cells is represented by the black histograms. Dotted histograms show staining with an isotype control antibody. Assays are representative of two independent experiments. (**d**) Analysis of E3 protein binding to LILRB1 and LILRB2 by SPR. LILRB1 and LILRB2 ECD were immobilized on sensor chips and binding of the indicated viral proteins, injected at 200 nM, was analyzed. In the case of the HAdV-F41 CR1β protein, the viral protein was immobilized on a sensor chip and LILRB1 and LILRB2 ECDs were run as analytes (200 nM). Positive binders have been labeled on the plots for ease of visualization.

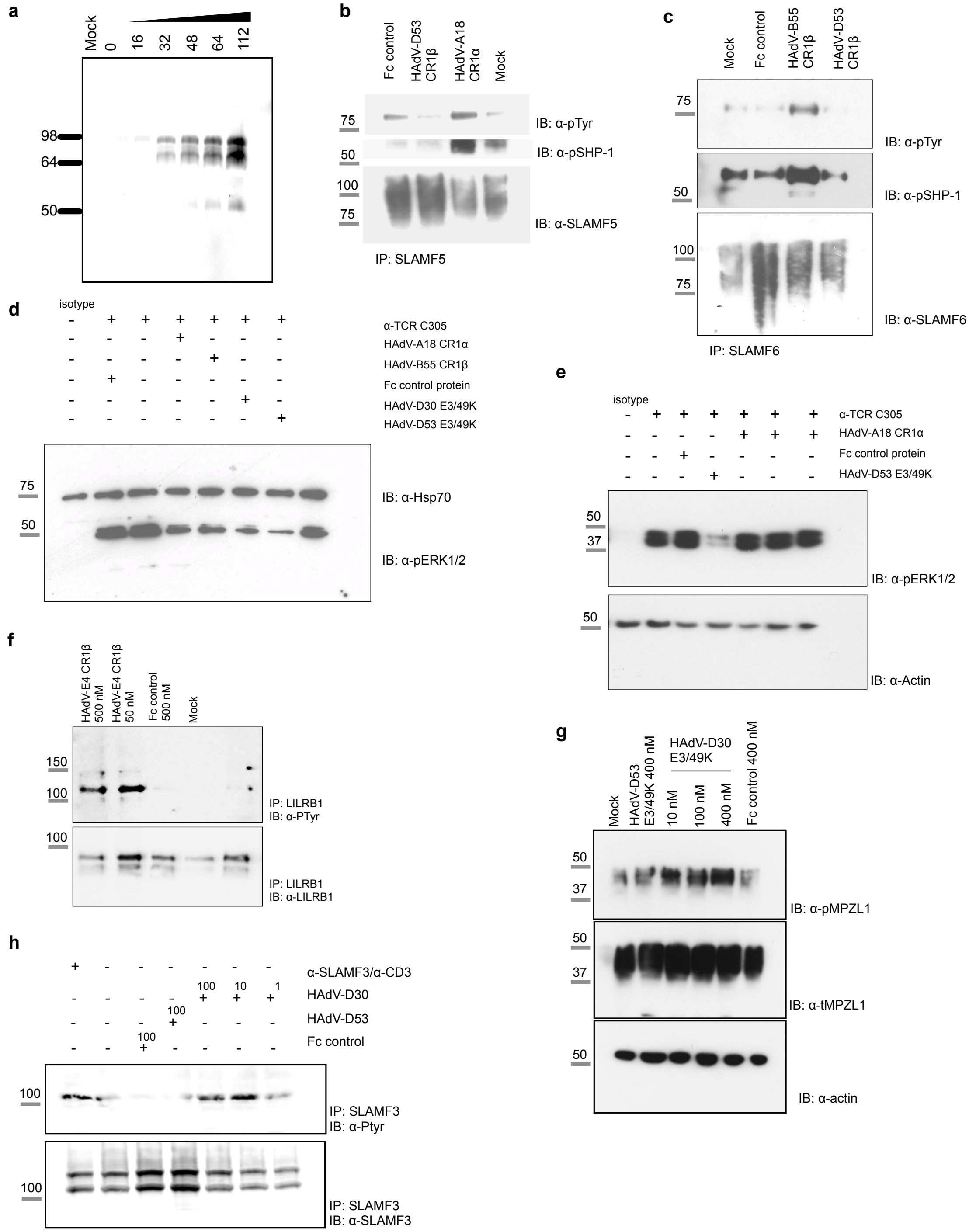


**Supplementary Figure 8. Full immunoblots corresponding to the assays shown throughout the manuscript.** (**a**) Analysis of E3/49K secretion from HAdV-D28-infected cells. Related to Fig. 3 (**b**) Receptor activation in SLAMF5 immunoprecipitates after stimulation with the indicated viral proteins (related to Fig. 4). (**c**) Receptor activation in SLAMF6 immunoprecipitates after stimulation with the indicated viral proteins (related to Fig. 4). (**d**) Analysis of ERK1/2

activation in Jurkat cells upon cell stimulation with an anti-TCR antibody and the indicated viral proteins (related to Fig. 4). Last lane (unlabeled) shows an additional protein not studied in the current work. (**e**) ERK1/2 activation in SLAMF5 KD Jurkat cells upon stimulation with the indicate proteins and an anti-TCR antibody (related to Fig. 4). (**f**) Receptor activation in LILRB1 immunoprecipitates upon stimulation with the indicated viral proteins. Last lane (unlabeled) shows an additional protein not studied in the current work (related to Fig. 5). (**g**) Activation of the receptor MPZL1 upon stimulation with the indicated viral proteins (related to Fig. 7). (**h**) SLAMF3 activation upon stimulation with the indicated viral proteins analyzed in receptor immunoprecipitates (related to Fig. 7). Molecular sizes are indicated in KDa.

**Supplementary Table 1. List of the proteins included in the Microarrays and their corresponding UniProt ID.**

| Gene name | UniProt ID | Gene name | UniProt ID | Gene name | UniProt ID |
|---|---|---|---|---|---|
| **A2M** | P01023 | **ANGPTL4** | Q9BY76 | **BGLAP** | P02818 |
| **ABHD14A** | Q9BUJ0 | **ANGPTL6** | Q8NI99 | **BGN** | P21810 |
| **ABP1** | P19801 | **ANGPTL7** | O43827 | **BMP10** | O95393 |
| **ACE2** | Q9BYF1 | **ANTXR1** | Q9H6X2 | **BMP15** | O95972 |
| **ACP6** | Q9NPH0 | **APCDD1** | Q8J025 | **BMP6** | P22004 |
| **ACRV1** | P26436 | **APCS** | P02743 | **BMPR1A** | P36894 |
| **ACVR1** | Q04771 | **APLP1** | P51693 | **BMPR1B** | O00238 |
| **ACVR1B** | P36896 | **APOA1** | P02647 | **BMPR2** | Q13873 |
| **ACVR1C** | Q8NER5 | **APOA2** | P02652 | **BOC** | Q9BWV1 |
| **ACVRL1** | P37023 | **APOA4** | P06727 | **BOLA1** | Q9Y3E2 |
| **ADAM10** | O14672 | **APOC1** | P02654 | **BPI** | P17213 |
| **ADAM11** | O75078 | **APOC2** | P02655 | **BPIFA2** | Q96DR5 |
| **ADAM12** | O43184 | **APOD** | P05090 | **BPIFB1** | Q8TDL5 |
| **ADAM15** | Q13444 | **APOE** | P02649 | **BPIFB2** | Q8N4F0 |
| **ADAMTSL1** | Q8N6G6 | **APOF** | Q13790 | **BSDC1** | Q9NW68 |
| **ADAMTSL5** | Q6ZMM2 | **APOH** | P02749 | **BSG** | P35613 |
| **ADCYAP1** | P18509 | **APP** | P05067 | **BST1** | Q10588 |
| **ADIPOQ** | Q15848 | **AREG** | P15514 | **BST2** | Q10589 |
| **ADM** | P35318 | **ART3** | Q13508 | **BTD** | P43251 |
| **AFM** | P43652 | **ART4** | Q93070 | **BTLA** | Q7Z6A9 |
| **AGER** | Q15109 | **ART5** | Q96L15 | **BTN1A1** | Q13410 |
| **AGR2** | O95994 | **ARTN** | Q5T4W7 | **BTN2A1** | Q7KYR7 |
| **AGR3** | Q8TD06 | **ASAH1** | Q13510 | **BTN2A2** | Q8WVV5 |
| **AGT** | P01019 | **ASGR1** | P07306 | **BTN2A3P** | Q96KV6 |
| **AHSG** | P02765 | **ASGR2** | P07307 | **BTN3A1** | O00481 |
| **AIF1L** | Q9BQI0 | **ASIP** | P42127 | **BTN3A2** | P78410 |
| **ALB** | P02768 | **ASPN** | Q9BXN1 | **BTN3A3** | O00478 |
| **ALCAM** | Q13740 | **ASTL** | Q6HA08 | **BTNL8** | Q6UX41 |
| **AMBN** | Q9NP70 | **ATP1B1** | P05026 | **BTNL9** | Q6UXG8 |
| **AMBP** | P02760 | **ATRAID** | Q6UW56 | **C10orf54** | Q9H7M9 |
| **AMD1** | P17707 | **AXL** | P30530 | **C10orf99** | Q6UWK7 |
| **AMELX** | Q99217 | **AZGP1** | P25311 | **C11orf24** | Q96F05 |
| **AMICA1** | Q86YT9 | **B2M** | P61769 | **C11orf68** | Q9H3H3 |
| **AMIGO2** | Q86SJ2 | **B3GNT1** | O43505 | **C11orf83** | Q6UW78 |
| **AMTN** | Q6UX39 | **B4GALT7** | Q9UBV7 | **C12orf39** | Q9BT56 |
| **AMY1A** | P04745 | **BAMBI** | Q13145 | **C12orf53** | Q8IYJ0 |
| **AMY1C** | P04745 | **BCAM** | P50895 | **C14orf37** | Q86TY3 |
| **AMY2B** | P19961 | **BCAN** | Q96GW7 | **C15orf24** | Q9NPA0 |
| **ANG** | P03950 | **BCHE** | P06276 | **C17orf99** | Q6UX52 |
| **ANGPTL2** | Q9UKU9 | **BCL10** | O95999 | **C19orf18** | Q8NEA5 |
| **ANGPTL3** | Q9Y5C1 | **BDNF** | P23560 | **C19orf38** | A8MVS5 |

| | | | | | |
|---|---|---|---|---|---|
| **C19orf59** | Q8IX19 | **CBLN4** | Q9NTU7 | **CD244** | Q9BZW8 |
| **C19orf63** | Q5UCC4 | **CCDC104** | Q96G28 | **CD27** | P26842 |
| **C1orf159** | Q96HA4 | **CCDC126** | Q96EE4 | **CD274** | Q9NZQ7 |
| **C1orf186** | Q6ZWK4 | **CCDC134** | Q9H6E4 | **CD276** | Q5ZPR3 |
| **C1orf187** | Q8NBI3 | **CCDC47** | Q96A33 | **CD28** | P10747 |
| **C1orf198** | Q9H425 | **CCDC80** | Q76M96 | **CD300A** | Q9UGN4 |
| **C1orf43** | Q9BWL3 | **CCK** | P06307 | **CD300C** | Q08708 |
| **C1orf85** | Q8WWB7 | **CCL1** | P22362 | **CD300E** | Q496F6 |
| **C1QA** | P02745 | **CCL11** | P51671 | **CD300LB** | A8K4G0 |
| **C1QB** | P02746 | **CCL13** | Q99616 | **CD300LD** | Q6UXZ3 |
| **C1QTNF3** | Q9BXJ4 | **CCL14** | Q16627 | **CD300LF** | Q8TDQ1 |
| **C1QTNF9** | P0C862 | **CCL16** | O15467 | **CD300LG** | Q6UXG3 |
| **C1QTNF9B** | B2RNN3 | **CCL17** | Q92583 | **CD302** | Q8IX05 |
| **C1R** | P00736 | **CCL18** | P55774 | **CD320** | Q9NPF0 |
| **C1RL** | Q9NZP8 | **CCL19** | Q99731 | **CD33** | P20138 |
| **C1S** | P09871 | **CCL2** | P13500 | **CD3D** | P04234 |
| **C20orf3** | Q9HDC9 | **CCL20** | P78556 | **CD3E** | P07766 |
| **C3** | P01024 | **CCL21** | O00585 | **CD3G** | P09693 |
| **C3orf64** | Q5NDL2 | **CCL22** | O00626 | **CD4** | P01730 |
| **C4BPB** | P20851 | **CCL24** | O00175 | **CD40** | P25942 |
| **C4orf26** | Q17RF5 | **CCL25** | O15444 | **CD40LG** | P29965 |
| **C4orf34** | Q96QK8 | **CCL26** | Q9Y258 | **CD44** | P16070 |
| **C5orf15** | Q8NC54 | **CCL27** | Q9Y4X3 | **CD46** | P15529 |
| **C6orf15** | Q6UXA7 | **CCL28** | Q9NRJ3 | **CD47** | Q08722 |
| **C6orf162** | Q96KF7 | **CCL3** | P10147 | **CD48** | P09326 |
| **C6orf25** | O95866 | **CCL3L3** | P16619 | **CD5** | P06127 |
| **C6orf58** | Q6P5S2 | **CCL4** | P13236 | **CD52** | P31358 |
| **C6orf72** | Q9NU53 | **CCL4L2** | Q8NHW4 | **CD55** | P08174 |
| **C7** | P10643 | **CCL5** | P13501 | **CD58** | P19256 |
| **C8G** | P07360 | **CCL7** | P80098 | **CD59** | P13987 |
| **C9orf11** | Q9NQ60 | **CCL8** | P80075 | **CD5L** | O43866 |
| **CA11** | O75493 | **CD14** | P08571 | **CD68** | P34810 |
| **CA12** | O43570 | **CD164** | Q04900 | **CD69** | Q07108 |
| **CA14** | Q9ULX7 | **CD164L2** | Q6UWJ8 | **CD7** | P09564 |
| **CA4** | P22748 | **CD177** | Q8N6Q3 | **CD72** | P21854 |
| **CA6** | P23280 | **CD180** | Q99467 | **CD79B** | P40259 |
| **CA9** | Q16790 | **CD19** | P15391 | **CD80** | P33681 |
| **CADM1** | Q9BY67 | **CD1D** | P15813 | **CD83** | Q01151 |
| **CADM2** | Q8N3J6 | **CD2** | P06729 | **CD84** | Q9UIB8 |
| **CADM3** | Q8N126 | **CD200** | P41217 | **CD86** | P42081 |
| **CALU** | O43852 | **CD200R1** | Q8TD46 | **CD8A** | P01732 |
| **CAMP** | P49913 | **CD200R1L** | Q6Q8B3 | **CD8B** | P10966 |
| **CARTPT** | Q16568 | **CD207** | Q9UJ71 | **CD93** | Q9NPY3 |
| **CBLN2** | Q8IUK8 | **CD226** | Q15762 | **CD96** | P40200 |
| **CBLN3** | Q6UW01 | **CD24** | P25063 | **CD99** | P14209 |

| | | | | | |
|---|---|---|---|---|---|
| **CD99L2** | Q8TCZ2 | **CFP** | P27918 | **CNTNAP2** | Q9UHC6 |
| **CDAN1** | Q8IWY9 | **CGA** | P01215 | **CNTNAP4** | Q9C0A0 |
| **CDCP1** | Q9H5V8 | **CGB1** | A6NKQ9 | **CNTNAP5** | Q8WYK1 |
| **CDH1** | P12830 | **CHAD** | O15335 | **COCH** | O43405 |
| **CDH11** | P55287 | **CHGA** | P10645 | **COL15A1** | P39059 |
| **CDH12** | P55289 | **CHGB** | P05060 | **COL1A2** | P08123 |
| **CDH13** | P55290 | **CHI3L1** | P36222 | **COL3A1** | P02461 |
| **CDH16** | O75309 | **CHI3L2** | Q15782 | **COL4A5** | P29400 |
| **CDH17** | Q12864 | **CHIA** | Q9BZP6 | **COL8A2** | P25067 |
| **CDH18** | Q13634 | **CHIT1** | Q13231 | **COL9A1** | P20849 |
| **CDH19** | Q9H159 | **CHL1** | O00533 | **COLEC10** | Q9Y6Z7 |
| **CDH20** | Q9HBT6 | **CHODL** | Q9H9P2 | **COLEC11** | Q9BWP8 |
| **CDH24** | Q86UP0 | **CHRDL2** | Q6WN34 | **COLEC12** | Q5KU26 |
| **CDH3** | P22223 | **CHST14** | Q8NCH0 | **COMP** | P49747 |
| **CDH4** | P55283 | **CLDN2** | P57739 | **CORT** | O00230 |
| **CDH5** | P33151 | **CLEC10A** | Q8IUN9 | **CPA1** | P15085 |
| **CDH6** | P55285 | **CLEC12A** | Q5QGZ9 | **CPA2** | P48052 |
| **CDH8** | P55286 | **CLEC12B** | Q2HXU8 | **CPA4** | Q9UI42 |
| **CDH9** | Q9ULB4 | **CLEC17A** | Q6ZS10 | **CPB1** | P15086 |
| **CDHR1** | Q96JP9 | **CLEC1A** | Q8NC01 | **CPB2** | Q96IY4 |
| **CDHR2** | Q9BYE9 | **CLEC1B** | Q9P126 | **CPQ** | Q9Y646 |
| **CDHR5** | Q9HBB8 | **CLEC2A** | Q6UVW9 | **CR2** | P20023 |
| **CDON** | Q4KMG0 | **CLEC2B** | Q92478 | **CRB1** | P82279 |
| **CDSN** | Q15517 | **CLEC2D** | Q9UHP7 | **CREG1** | O75629 |
| **CEACAM1** | P13688 | **CLEC2L** | P0C7M8 | **CRELD1** | Q96HD1 |
| **CEACAM19** | Q7Z692 | **CLEC3B** | P05452 | **CRELD2** | Q6UXH1 |
| **CEACAM20** | Q6UY09 | **CLEC4A** | Q9UMR7 | **CRHBP** | P24387 |
| **CEACAM21** | Q3KPI0 | **CLEC4D** | Q8WXI8 | **CRIM1** | Q9NZV1 |
| **CEACAM3** | P40198 | **CLEC4E** | Q9ULY5 | **CRISP3** | P54108 |
| **CEACAM4** | O75871 | **CLEC5A** | Q9NY25 | **CRISPLD2** | Q9H0B8 |
| **CEACAM5** | P06731 | **CLEC7A** | Q9BXN2 | **CRP** | P02741 |
| **CEACAM6** | P40199 | **CLEC9A** | Q6UXN8 | **CRTAM** | O95727 |
| **CEACAM7** | Q14002 | **CLMP** | Q9H6B4 | **CSF1** | P09603 |
| **CEACAM8** | P31997 | **CLPS** | P04118 | **CSF1R** | P07333 |
| **CECR1** | Q9NZK5 | **CLSTN3** | Q9BQT9 | **CSF2** | P04141 |
| **CELA2A** | P08217 | **CLU** | P10909 | **CSF2RA** | P15509 |
| **CELA3A** | P09093 | **CLUL1** | Q15846 | **CSF2RB** | P32927 |
| **CER1** | O95813 | **CNDP1** | Q96KN2 | **CSF3** | P09919 |
| **CES3** | Q6UWW8 | **CNIH** | B2R4P1 | **CSF3R** | Q99062 |
| **CFB** | P00751 | **CNPY3** | Q9BT09 | **CSH2** | P0DML3 |
| **CFHR1** | Q03591 | **CNPY4** | Q8N129 | **CSN2** | P05814 |
| **CFHR2** | P36980 | **CNTN2** | Q02246 | **CSN3** | P07498 |
| **CFHR3** | Q02985 | **CNTN3** | Q9P232 | **CSPG5** | O95196 |
| **CFHR5** | Q9BXR6 | **CNTN5** | O94779 | **CSRP2BP** | Q9H8E8 |
| **CFI** | P05156 | **CNTN6** | Q9UQ52 | **CST1** | P01037 |

| | | | | | |
|---|---|---|---|---|---|
| **CST2** | P09228 | **DEFB1** | P60022 | **EFNA4** | P52798 |
| **CST3** | P01034 | **DEFB104A** | Q8WTQ1 | **EFNB1** | P98172 |
| **CST4** | P01036 | **DEFB105A** | Q8NG35 | **EFNB2** | P52799 |
| **CST5** | P28325 | **DEFB106A** | Q8N104 | **EFNB3** | Q15768 |
| **CST6** | Q15828 | **DEFB108B** | Q8NET1 | **EGF** | P01133 |
| **CST7** | O76096 | **DEFB110** | Q30KQ9 | **EGFL7** | Q9UHF1 |
| **CST9L** | Q9H4G1 | **DEFB113** | Q30KQ7 | **EGFLAM** | Q63HQ2 |
| **CTGF** | P29279 | **DEFB114** | Q30KQ6 | **EGFR** | P00533 |
| **CTHRC1** | Q96CG8 | **DEFB118** | Q96PH6 | **ELN** | P15502 |
| **CTLA4** | P16410 | **DEFB119** | Q8N690 | **ELTD1** | Q9HBW9 |
| **CTRB2** | Q6GPI1 | **DEFB121** | Q5J5C9 | **EMB** | Q6PCB8 |
| **CTRC** | Q99895 | **DEFB127** | Q9H1M4 | **EMCN** | Q9ULC0 |
| **CTRL** | P40313 | **DEFB132** | Q7Z7B7 | **EMR3** | Q9BY15 |
| **CTSL2** | O60911 | **DEFB136** | Q30KP8 | **ENDOU** | P21128 |
| **CX3CL1** | P78423 | **DEFB4A** | O15263 | **ENPEP** | Q07075 |
| **CXADR** | P78310 | **DHH** | O43323 | **ENPP5** | Q9UJA9 |
| **CXCL1** | P09341 | **DHRS9** | Q9BPW9 | **ENPP6** | Q6UWR7 |
| **CXCL10** | P02778 | **DKK2** | Q9UBU2 | **ENPP7** | Q6UWV6 |
| **CXCL11** | O14625 | **DKK3** | Q9UBP4 | **EPCAM** | P16422 |
| **CXCL12** | P48061 | **DKK4** | Q9UBT3 | **EPGN** | Q6UW88 |
| **CXCL14** | O95715 | **DKKL1** | Q9UK85 | **EPHA2** | P29317 |
| **CXCL15** | Q9WVL7 | **DLK1** | P80370 | **EPHA3** | P29320 |
| **CXCL16** | Q9H2A7 | **DLL1** | O00548 | **EPHA4** | P54764 |
| **CXCL17** | Q6UXB2 | **DLL4** | Q9NR61 | **EPHA5** | P54756 |
| **CXCL2** | P19875 | **DNASE1** | P24855 | **EPHA6** | Q9UF33 |
| **CXCL3** | P19876 | **DPEP1** | P16444 | **EPHA7** | Q15375 |
| **CXCL5** | P42830 | **DPP4** | P27487 | **EPHB1** | P54762 |
| **CXCL6** | P80162 | **DPP6** | P42658 | **EPHB2** | P29323 |
| **CXCL9** | Q07325 | **DSC1** | Q08554 | **EPHB3** | P54753 |
| **CXorf61** | Q5H943 | **DSC2** | Q02487 | **EPHB4** | P54760 |
| **CYR61** | O00622 | **DSCAM** | O60469 | **EPHB6** | O15197 |
| **CYTL1** | Q9NRR1 | **DSG1** | Q02413 | **EPO** | P01588 |
| **CYYR1** | Q96J86 | **DSG2** | Q14126 | **EPOR** | P19235 |
| **DAG1** | Q14118 | **DSG4** | Q86SJ6 | **ERBB2** | P04626 |
| **DCC** | P43146 | **ECE1** | P42892 | **EREG** | O14944 |
| **DCD** | P81605 | **ECM1** | Q16610 | **ERMAP** | Q96PL5 |
| **DCN** | P07585 | **EDA2R** | Q9HAV5 | **ERO1L** | Q96HE7 |
| **DCT** | P40126 | **EDAR** | Q9UNE0 | **ERP27** | Q96DN0 |
| **DDOST** | P39656 | **EDDM3B** | P56851 | **ERP44** | Q9BS26 |
| **DDR1** | Q08345 | **EDIL3** | O43854 | **ESAM** | Q96AP7 |
| **DDR2** | Q16832 | **EDN2** | P20800 | **ESM1** | Q9NQ30 |
| **DEFA3** | P59666 | **EDN3** | P14138 | **ESYT3** | A0FGR9 |
| **DEFA4** | P12838 | **EEF1D** | P29692 | **EVI2A** | P22794 |
| **DEFA5** | Q01523 | **EFNA1** | P20827 | **EVI2B** | P34910 |
| **DEFA6** | Q01524 | **EFNA3** | P52797 | **F10** | P00742 |

| | | | | | |
|---|---|---|---|---|---|
| **F11** | P03951 | **FGF12** | P61328 | **GAL** | P22466 |
| **F11R** | Q9Y624 | **FGF13** | Q92913 | **GALP** | Q9UBC7 |
| **F3** | P13726 | **FGF14** | Q92915 | **GC** | P02774 |
| **F9** | P00740 | **FGF17** | O60258 | **GDF15** | Q99988 |
| **FAIM3** | O60667 | **FGF18** | O76093 | **GDF5** | P43026 |
| **FAM150B** | Q6UX46 | **FGF19** | O95750 | **GDF9** | O60383 |
| **FAM151A** | Q8WW52 | **FGF21** | Q9NSA1 | **GDNF** | P39905 |
| **FAM159A** | Q6UWV7 | **FGF22** | Q9HCT0 | **GFRA1** | P56159 |
| **FAM171A1** | Q5VUB5 | **FGF23** | Q9GZV9 | **GFRA3** | O60609 |
| **FAM171B** | Q6P995 | **FGF6** | P10767 | **GFRAL** | Q6UXV0 |
| **FAM174A** | Q8TBP5 | **FGF8** | P55075 | **GGT1** | P19440 |
| **FAM180A** | Q6UWF9 | **FGF9** | P31371 | **GH1** | P01241 |
| **FAM187B** | Q17R55 | **FGFBP1** | Q14512 | **GH2** | P01242 |
| **FAM189A2** | Q15884 | **FGFBP2** | Q9BYJ0 | **GHR** | P10912 |
| **FAM19A1** | Q7Z5A9 | **FGFBP3** | Q8TAT2 | **GIF** | P27352 |
| **FAM19A2** | Q8N3H0 | **FGFR1** | P11362 | **GKN1** | Q9NS71 |
| **FAM19A3** | Q7Z5A8 | **FGFR2** | P21802 | **GKN2** | Q86XP6 |
| **FAM19A4** | Q96LR4 | **FGFR3** | P22607 | **GNLY** | P22749 |
| **FAM19A5** | Q7Z5A7 | **FGFR4** | P22455 | **GNRH1** | P01148 |
| **FAM209A** | Q5JX71 | **FGFRL1** | Q8N441 | **GNRH2** | O43555 |
| **FAM209B** | Q5JX69 | **FGL1** | Q08830 | **GP1BA** | P07359 |
| **FAM3B** | P58499 | **FIBCD1** | Q8N539 | **GPA33** | Q99795 |
| **FAM3D** | Q96BQ1 | **FKBP11** | Q9NYL4 | **GPC2** | Q8N158 |
| **FAS** | P25445 | **FKBP14** | Q9NWM8 | **GPC3** | P51654 |
| **FASLG** | P48023 | **FKBP7** | Q9Y680 | **GPC6** | Q9Y625 |
| **FCAMR** | Q8WWV6 | **FLRT1** | Q9NZU1 | **GPHA2** | Q96T91 |
| **FCER2** | P06734 | **FLRT2** | O43155 | **GPIHBP1** | Q8IV16 |
| **FCGR2A** | P12318 | **FLRT3** | Q9NZU0 | **GPLD1** | P80108 |
| **FCGR2B** | P31994 | **FLT1** | P17948 | **GPR114** | Q8IZF4 |
| **FCGR3B** | O75015 | **FLT3LG** | P49771 | **GPR56** | Q9Y653 |
| **FCGRT** | P55899 | **FLT4** | P35916 | **GPR97** | Q86Y34 |
| **FCN1** | O00602 | **FN1** | P02751 | **GPX7** | Q96SL4 |
| **FCN2** | Q15485 | **FNDC4** | Q9H6D8 | **GPX8** | Q8TED1 |
| **FCN3** | O75636 | **FOLR1** | P15328 | **GREM1** | O60565 |
| **FCRL1** | Q96LA6 | **FOLR2** | P14207 | **GREM2** | Q9H772 |
| **FCRL2** | Q96LA5 | **FOLR3** | P41439 | **GRN** | P28799 |
| **FCRL3** | Q96P31 | **FRZB** | Q92765 | **GSG1** | Q2KHT4 |
| **FCRL4** | Q96PJ5 | **FSHB** | P01225 | **GUCA2A** | Q02747 |
| **FCRL5** | Q96RD9 | **FST** | P19883 | **GUCA2B** | Q16661 |
| **FCRL6** | Q6DN72 | **FSTL1** | Q12841 | **GUCY2C** | P25092 |
| **FCRLA** | Q7L513 | **FSTL3** | O95633 | **GYPA** | P02724 |
| **FDCSP** | Q8NFU4 | **FSTL5** | Q8N475 | **GYPC** | P04921 |
| **FETUB** | Q9UGM5 | **FXYD4** | P59646 | **GZMA** | P12544 |
| **FGF1** | P05230 | **FXYD5** | Q96DB9 | **GZMB** | P10144 |
| **FGF10** | O15520 | **FZD2** | Q14332 | **HAPLN4** | Q86UW8 |

| | | | | | |
|---|---|---|---|---|---|
| **HAVCR1** | Q96D42 | **IGF1** | P05019 | **IL1RL1** | Q01638 |
| **HAVCR2** | Q8TDQ0 | **IGF2** | P01344 | **IL1RL2** | Q9HB29 |
| **HBEGF** | Q99075 | **IGFBP1** | P08833 | **IL2** | P60568 |
| **HEPACAM** | Q14CZ8 | **IGFBP3** | P17936 | **IL20** | Q9NYY1 |
| **HEPACAM2** | A8MVW5 | **IGFBP4** | P22692 | **IL20RA** | Q9UHF4 |
| **HFE** | Q30201 | **IGFBP5** | P24593 | **IL20RB** | Q6UXL0 |
| **HFE2** | Q6ZVN8 | **IGFBP6** | P24592 | **IL21** | Q9HBE4 |
| **HGF** | P14210 | **IGFBP7** | Q16270 | **IL21R** | Q9HBE5 |
| **HHIP** | Q96QV1 | **IGFL1** | Q6UW32 | **IL22** | Q9GZX6 |
| **HHLA2** | Q9UM44 | **IGFL3** | Q6UXB1 | **IL22RA2** | Q969J5 |
| **HPR** | P00739 | **IGFL4** | Q6B9Z1 | **IL23R** | Q5VWK5 |
| **HRG** | P04196 | **IGFLR1** | Q9H665 | **IL24** | Q13007 |
| **HTN3** | P15516 | **IGSF11** | Q5DX21 | **IL25** | Q9H293 |
| **HTRA3** | P83110 | **IGSF22** | Q8N9C0 | **IL27RA** | Q6UWB1 |
| **HTRA4** | P83105 | **IGSF6** | O95976 | **IL28A** | Q8IZJ0 |
| **HYAL1** | Q12794 | **IGSF8** | Q969P0 | **IL29** | Q8IU54 |
| **HYAL2** | Q12891 | **IL10** | P22301 | **IL2RB** | P14784 |
| **IAPP** | P10997 | **IL10RA** | Q13651 | **IL3** | P08700 |
| **IBSP** | P21815 | **IL10RB** | Q08334 | **IL31RA** | Q8NI17 |
| **ICAM1** | P05362 | **IL11** | P20809 | **IL33** | O95760 |
| **ICAM2** | P13598 | **IL12B** | P29460 | **IL36A** | Q9UHA7 |
| **ICAM3** | P32942 | **IL13RA2** | Q14627 | **IL36G** | Q9NZH8 |
| **ICOS** | Q9Y6W8 | **IL15** | P40933 | **IL36RN** | Q9UBH0 |
| **ICOSLG** | O75144 | **IL17A** | Q16552 | **IL4** | P05112 |
| **IFI30** | P13284 | **IL17B** | Q9UHF5 | **IL4I1** | Q96RQ9 |
| **IFNA1** | P01562 | **IL17C** | Q9P0M4 | **IL4R** | P24394 |
| **IFNA10** | P01566 | **IL17D** | Q8TAD2 | **IL5** | P05113 |
| **IFNA13** | P01562 | **IL17F** | Q96PD4 | **IL6** | P05231 |
| **IFNA14** | P01570 | **IL17RA** | Q96F46 | **IL6ST** | P40189 |
| **IFNA16** | P05015 | **IL17RB** | Q9NRM6 | **IL7** | P13232 |
| **IFNA17** | P01571 | **IL17RC** | Q8NAC3 | **IL8** | P10145 |
| **IFNA2** | P01563 | **IL17RD** | Q8NFM7 | **IL9** | P15248 |
| **IFNA21** | P01568 | **IL18** | Q14116 | **ILDR2** | Q71H61 |
| **IFNA4** | P05014 | **IL18BP** | O95998 | **INHBC** | P55103 |
| **IFNA5** | P01569 | **IL18R1** | Q13478 | **INSL3** | P51460 |
| **IFNA6** | P05013 | **IL18RAP** | O95256 | **INSL4** | Q14641 |
| **IFNA7** | P01567 | **IL19** | Q9UHD0 | **INSL5** | Q9Y5Q6 |
| **IFNA8** | P32881 | **IL1A** | P01583 | **INSRR** | P14616 |
| **IFNAR1** | P17181 | **IL1B** | P01584 | **ISLR** | O14498 |
| **IFNAR2** | P48551 | **IL1F10** | Q8WWZ1 | **ISLR2** | Q6UXK2 |
| **IFNB1** | P01574 | **IL1R1** | P14778 | **ISM2** | Q6H9L7 |
| **IFNG** | P01579 | **IL1R2** | P27930 | **ITFG1** | Q8TB96 |
| **IFNGR2** | P38484 | **IL1RAP** | Q9NPH3 | **ITGA10** | O75578 |
| **IFNW1** | P05000 | **IL1RAPL1** | Q9NZN1 | **ITGA5** | P08648 |
| **IGDCC3** | Q8IVU1 | **IL1RAPL2** | Q9NP60 | **ITGB6** | P18564 |

| | | | | | |
|---|---|---|---|---|---|
| **ITIH1** | P19827 | **KLRB1** | Q12918 | **LRFN2** | Q9ULH4 |
| **ITM2A** | O43736 | **KLRC1** | P26715 | **LRFN5** | Q96NI6 |
| **ITM2B** | Q9Y287 | **KLRC2** | P26717 | **LRG1** | P02750 |
| **ITM2C** | Q9NQX7 | **KLRD1** | Q13241 | **LRIG1** | Q96JA1 |
| **IZUMO1** | Q8IYV9 | **KLRF1** | Q9NZS2 | **LRIG2** | O94898 |
| **IZUMO4** | Q1ZYL8 | **KLRG1** | Q96E93 | **LRIG3** | Q6UXM1 |
| **JAG1** | P78504 | **KLRK1** | P26718 | **LRP10** | Q7Z4F1 |
| **JAM2** | P57087 | **KNG1** | P01042 | **LRPAP1** | P30533 |
| **JAM3** | Q9BX67 | **L1CAM** | P32004 | **LRRC15** | Q8TF66 |
| **KAZALD1** | Q96I82 | **LACRT** | Q9GZZ8 | **LRRC19** | Q9H756 |
| **KDELC1** | Q6UW63 | **LAIR1** | Q6GTX8 | **LRRC25** | Q8N386 |
| **KDR** | P35968 | **LAIR2** | Q6ISS4 | **LRRC3** | Q9BY71 |
| **KERA** | O60938 | **LAMP2** | P13473 | **LRRC32** | Q14392 |
| **KIAA0319L** | Q8IZA0 | **LAMP3** | Q9UQV4 | **LRRC37A2** | A6NM11 |
| **KIAA1467** | A2RU67 | **LAMP5** | Q9UJQ1 | **LRRC37B** | Q96QE4 |
| **KIR2DL1** | P43626 | **LAYN** | Q6UX15 | **LRRC3B** | Q96PB8 |
| **KIR2DL3** | P43628 | **LBP** | P18428 | **LRRC4** | Q9HBW1 |
| **KIR2DL4** | Q99706 | **LCAT** | P04180 | **LRRC4C** | Q9HCJ2 |
| **KIR2DL5A** | Q8N109 | **LCN1** | P31025 | **LRRC52** | Q8N7C0 |
| **KIR2DL5B** | Q8NHK3 | **LCTL** | Q6UWM7 | **LRRN1** | Q6UXK5 |
| **KIR2DS1** | Q14954 | **LDL** | | **LRRN4** | Q8WUT4 |
| **KIR2DS4** | P43632 | **LDLR** | P01130 | **LRRN4CL** | Q8ND94 |
| **KIR2DS5** | Q14953 | **LDLRAD3** | Q86YD5 | **LRRTM1** | Q86UE6 |
| **KIR3DL1** | P43629 | **LECT1** | O75829 | **LRRTM2** | O43300 |
| **KIR3DL2** | P43630 | **LEFTY1** | O75610 | **LRRTM3** | Q86VH5 |
| **KIR3DL3** | Q8N743 | **LEFTY2** | O00292 | **LRRTM4** | Q86VH4 |
| **KIR3DX1** | Q9H7L2 | **LEP** | P41159 | **LRTM1** | Q9HBL6 |
| **KIRREL** | Q96J84 | **LEPR** | P48357 | **LRTM2** | Q8N967 |
| **KIRREL2** | Q6UWL6 | **LIF** | P15018 | **LSAMP** | Q13449 |
| **KIRREL3** | Q8IZU9 | **LIFR** | P42702 | **LSR** | Q86X29 |
| **KISS1** | Q15726 | **LILRA1** | O75019 | **LTA** | P01374 |
| **KIT** | P10721 | **LILRA4** | P59901 | **LTB** | Q06643 |
| **KITLG** | P21583 | **LILRA5** | A6NI73 | **LTBR** | P36941 |
| **KLK1** | P06870 | **LILRA6** | Q6PI73 | **LUM** | P51884 |
| **KLK10** | O43240 | **LILRB1** | Q8NHL6 | **LUZP2** | Q86TE4 |
| **KLK11** | Q9UBX7 | **LILRB2** | Q8N423 | **LY6D** | Q14210 |
| **KLK12** | Q9UKR0 | **LILRB4** | Q8NHJ6 | **LY6G6C** | O95867 |
| **KLK13** | Q9UKR3 | **LILRB5** | O75023 | **LY6G6D** | O95868 |
| **KLK3** | P07288 | **LINGO2** | Q7L985 | **LY6G6F** | Q5SQ64 |
| **KLK4** | Q9Y5K2 | **LIPG** | Q9Y5X9 | **LY6H** | O94772 |
| **KLK5** | Q9Y337 | **LIPH** | Q8WWY8 | **LY6K** | Q17RY6 |
| **KLK6** | Q92876 | **LMAN1** | P49257 | **LY86** | O95711 |
| **KLK7** | P49862 | **LOXL2** | Q9Y4K0 | **LY9** | Q9HBG7 |
| **KLK8** | O60259 | **LPAL2** | Q16609 | **LYG1** | Q8N1E2 |
| **KLK9** | Q9UKQ9 | **LPL** | P06858 | **LYG2** | Q86SG7 |

| | | | | | |
|---|---|---|---|---|---|
| **LYNX1** | Q9BZG9 | **MMP1** | P03956 | **NETO2** | Q8NC67 |
| **LYPD2** | Q6UXB3 | **MMP10** | P09238 | **NFAM1** | Q8NET5 |
| **LYPD3** | O95274 | **MMP12** | P39900 | **NFASC** | O94856 |
| **LYPD5** | Q6UWN5 | **MMP13** | P45452 | **NGF** | P01138 |
| **LYPD6** | Q86Y78 | **MMP15** | P51511 | **NGFR** | P08138 |
| **LYPD6B** | Q8NI32 | **MMP16** | P51512 | **NID2** | Q14112 |
| **LYSMD3** | Q7Z3D4 | **MMP2** | P08253 | **NLGN1** | Q8N2Q7 |
| **LYVE1** | Q9Y5Y7 | **MMP20** | O60882 | **NLGN4X** | Q8N0W4 |
| **LYZ** | P61626 | **MMP7** | P09237 | **NMU** | P48645 |
| **LYZL2** | Q7Z4W2 | **MMP8** | P22894 | **NOG** | Q13253 |
| **LYZL4** | Q96KX0 | **MMP9** | P14780 | **NOV** | P48745 |
| **LYZL6** | O75951 | **MOG** | Q16653 | **NPC2** | P61916 |
| **MAG** | P20916 | **MPL** | P40238 | **NPDC1** | Q9NQX5 |
| **MAGEA4** | P43358 | **MPZ** | P25189 | **NPHS1** | O60500 |
| **MAGT1** | Q9H0U3 | **MPZL1** | O95297 | **NPPB** | P16860 |
| **MAN1B1** | Q9UKM7 | **MPZL2** | O60487 | **NPTN** | Q9Y639 |
| **MANF** | P55145 | **MPZL3** | Q6UWV2 | **NPY** | P01303 |
| **MANSC1** | Q9H8J5 | **MSLN** | Q13421 | **NRCAM** | Q92823 |
| **MBL2** | P11226 | **MSMB** | P08118 | **NRG1** | Q02297 |
| **MCAM** | P43121 | **MSR1** | P21757 | **NRG4** | Q8WWG1 |
| **MDA-LDL** | | **MSRA** | Q9UJ68 | **NRN1** | Q9NPD7 |
| **MDGA2** | Q7Z553 | **MSRB3** | Q8IXL7 | **NRN1L** | Q496H8 |
| **MDK** | P21741 | **MST1** | P26927 | **NRP2** | O60462 |
| **MEGF10** | Q96KG7 | **MST1R** | Q04912 | **NRTN** | Q99748 |
| **MEGF9** | Q9H1U4 | **MSTN** | O14793 | **NRXN1** | Q9ULB1 |
| **MEP1A** | Q16819 | **MUC15** | Q8N387 | **NT5E** | P21589 |
| **MEP1B** | Q16820 | **MUC20** | Q8N307 | **NTF3** | P20783 |
| **MESDC2** | Q14696 | **MUCL1** | Q96DR8 | **NTF4** | P34130 |
| **MET** | P08581 | **MXRA8** | Q9BRK3 | **NTM** | Q9P121 |
| **METTL7A** | Q9H8H3 | **MYOC** | Q99972 | **NTN1** | O95631 |
| **MFAP3** | P55082 | **MZB1** | Q8WU39 | **NTNG1** | Q9Y2I2 |
| **MFAP3L** | O75121 | **NAALAD2** | Q9Y3Q0 | **NTNG2** | Q96CW9 |
| **MFAP4** | P55083 | **NAPSA** | O96009 | **NTRK1** | P04629 |
| **MFAP5** | Q13361 | **NBL1** | P41271 | **NTRK2** | Q16620 |
| **MFGE8** | Q08431 | **NCAM1** | P13591 | **NTRK3** | Q16288 |
| **MFI2** | P08582 | **NCAM2** | O15394 | **NTS** | P30990 |
| **MFRP** | Q9BY79 | **NCR1** | O76036 | **NXPH1** | P58417 |
| **MGP** | P08493 | **NCR2** | O95944 | **NXPH2** | O95156 |
| **MIA** | Q16674 | **NCR3** | O14931 | **NXPH3** | O95157 |
| **MIA3** | Q5JRA6 | **NCSTN** | Q92542 | **NXPH4** | O95158 |
| **MICB** | Q29980 | **NDP** | Q00604 | **OBP2A** | Q9NY56 |
| **MILR1** | Q7Z6M3 | **NEGR1** | Q7Z3B1 | **OBP2B** | Q9NPH6 |
| **MINPP1** | Q9UNW1 | **NELL1** | Q92832 | **ODAM** | A1E959 |
| **MLN** | P12872 | **NELL2** | Q99435 | **OGN** | P20774 |
| **MME** | P08473 | **NETO1** | Q8TDF5 | **OLFM3** | Q96PB7 |

| | | | | | |
|---|---|---|---|---|---|
| **OLFM4** | Q6UX06 | **PGF** | P49763 | **PRAP1** | Q96NZ9 |
| **OMD** | Q99983 | **PGLYRP1** | O75594 | **PRELP** | P51888 |
| **OMG** | P23515 | **PI16** | Q6UXB8 | **PRG2** | P13727 |
| **OPCML** | Q14982 | **PI3** | P19957 | **PRG3** | Q9Y2Y8 |
| **ORM1** | P02763 | **PIGR** | P01833 | **PRH1** | P02810 |
| **ORM2** | P19652 | **PIK3IP1** | Q96FE7 | **PRH2** | P02810 |
| **OSM** | P13725 | **PILRA** | Q9UKJ1 | **PRL** | P01236 |
| **OSMR** | Q99650 | **PILRB** | Q9UKJ0 | **PRLR** | P16471 |
| **OSTM1** | Q86WC4 | **PIP** | P12273 | **PRND** | Q9UKY0 |
| **OTOL1** | A6NHN0 | **PLA2G10** | O15496 | **PRNP** | **P04156** |
| **OTOR** | Q9NRC9 | **PLA2G12A** | Q9BZM1 | **PROCR** | Q9UNN8 |
| **PAEP** | P09466 | **PLA2G12B** | Q9BX93 | **PROK1** | P58294 |
| **PARM1** | Q6UWI2 | **PLA2G15** | Q8NCC3 | **PROS1** | P07225 |
| **PATE1** | Q8WXA2 | **PLA2G1B** | P04054 | **PRRG3** | Q9BZD7 |
| **PCDH10** | Q9P2E7 | **PLA2G2A** | P14555 | **PRRG4** | Q9BZD6 |
| **PCDH11X** | Q9BZA7 | **PLA2G2D** | Q9UNK4 | **PRRT3** | Q5FWE3 |
| **PCDH11Y** | Q9BZA8 | **PLA2G2E** | Q9NZK7 | **PRSS1** | P07477 |
| **PCDH12** | Q9NPG4 | **PLA2G7** | Q13093 | **PRSS27** | Q9BQR3 |
| **PCDH17** | O14917 | **PLAC1L** | Q86WS3 | **PRSS37** | A4D1T9 |
| **PCDH18** | Q9HCL0 | **PLAC9** | Q5JTB6 | **PRSS42** | Q7Z5A4 |
| **PCDH19** | Q8TAB3 | **PLAT** | P00750 | **PRSS55** | Q6UWB4 |
| **PCDH9** | Q9HC56 | **PLAU** | P00749 | **PRSS58** | Q8IYP2 |
| **PCDHB1** | Q9Y5F3 | **PLAUR** | Q03405 | **PRSS8** | Q16651 |
| **PCDHB14** | Q9Y5E9 | **PLB1** | Q6P1J6 | **PRTG** | Q2VWP7 |
| **PCDHB4** | Q9Y5E5 | **PLG** | P00747 | **PRTN3** | P24158 |
| **PCDHB9** | Q9Y5E1 | **PLVAP** | Q9BX97 | **PSCA** | O43653 |
| **PCDHGA11** | Q9Y5H2 | **PLXDC1** | Q8IUK5 | **PSG1** | P11464 |
| **PCDHGA12** | O60330 | **PLXDC2** | Q6UX71 | **PSG11** | Q9UQ72 |
| **PCOLCE** | Q15113 | **PLXNB2** | O15031 | **PSG3** | Q16557 |
| **PCYOX1L** | Q8NBM8 | **PMCH** | P20382 | **PSG4** | Q00888 |
| **PDCD1** | Q15116 | **PMEL** | P40967 | **PSG5** | Q15238 |
| **PDCD1LG2** | Q9BQ51 | **PNLIP** | P16233 | **PSG6** | Q00889 |
| **PDGFA** | P04085 | **PNLIPRP2** | P54317 | **PSG7** | Q13046 |
| **PDGFC** | Q9NRA1 | **PNOC** | Q13519 | **PSG9** | Q00887 |
| **PDGFD** | Q9GZP0 | **PODN** | Q7Z5L7 | **PSPN** | O60542 |
| **PDGFRB** | P09619 | **PODXL** | O00592 | **PTGDS** | P41222 |
| **PDGFRL** | Q15198 | **PODXL2** | Q9NZ53 | **PTGFRN** | Q9P2B2 |
| **PDYN** | P01213 | **POGLUT1** | Q8NBL1 | **PTH** | P01270 |
| **PEA15** | Q15121 | **POMC** | P01189 | **PTHLH** | P12272 |
| **PEBP4** | Q96S96 | **POMGNT1** | Q8WZA1 | **PTK7** | Q13308 |
| **PECAM1** | P16284 | **PON1** | P27169 | **PTN** | P21246 |
| **PENK** | P01210 | **POSTN** | Q15063 | **PTPRA** | P18433 |
| **PF4** | P02776 | **PPBP** | P02775 | **PTPRC** | P08575 |
| **PGA3** | P0DJD8 | **PPY** | P01298 | **PTPRG** | P23470 |
| **PGC** | P20142 | **PRADC1** | Q9BSG0 | **PTPRH** | Q9HD43 |

| | | | | | |
|---|---|---|---|---|---|
| **PTPRK** | Q15262 | **SARAF** | Q96BY9 | **SERPINA12** | Q8IW75 |
| **PTPRM** | P28827 | **SBSN** | Q6UWP8 | **SERPINA3** | P01011 |
| **PTPRO** | Q16827 | **SCARB1** | Q8WTV0 | **SERPINA4** | P29622 |
| **PTPRR** | Q15256 | **SCG3** | Q8WXD2 | **SERPINA5** | P05154 |
| **PTPRT** | O14522 | **SCG5** | P05408 | **SERPINA6** | P08185 |
| **PVR** | P15151 | **SCGB1A1** | P11684 | **SERPINA7** | P05543 |
| **PVRIG** | Q6DKI7 | **SCGB1C1** | Q8TD33 | **SERPINA9** | Q86WD7 |
| **PVRL1** | Q15223 | **SCGB1D2** | O95969 | **SERPINB2** | P05120 |
| **PVRL2** | Q92692 | **SCGB1D4** | Q6XE38 | **SERPINC1** | P01008 |
| **PVRL3** | Q9NQS3 | **SCGB2A1** | O75556 | **SERPIND1** | P05546 |
| **PVRL4** | Q96NY8 | **SCGB2A2** | Q13296 | **SERPINE1** | P05121 |
| **PYY** | P10082 | **SCGB3A1** | Q96QR1 | **SERPINE2** | P07093 |
| **QPCT** | Q16769 | **SCGB3A2** | Q96PL1 | **SERPINF1** | P36955 |
| **QRFP** | P83859 | **SCN1B** | Q07699 | **SERPINF2** | P08697 |
| **RAET1E** | Q8TD07 | **SCN2B** | O60939 | **SERPING1** | P05155 |
| **RAET1L** | Q5VY80 | **SCN3B** | Q9NY72 | **SERPINI1** | Q99574 |
| **RARRES1** | P49788 | **SCN4B** | Q8IWT1 | **SERPINI2** | O75830 |
| **RARRES2** | Q99969 | **SCPEP1** | Q9HB40 | **SFRP1** | Q8N474 |
| **Rat Tail Collagen** | | **SCRG1** | O75711 | **SFRP2** | Q96HF1 |
| **RBP4** | P02753 | **SCT** | P09683 | **SFRP4** | Q6FHJ7 |
| **RCN3** | Q96D15 | **SDC1** | P18827 | **SFTA2** | Q6UW10 |
| **RECK** | O95980 | **SDC2** | P34741 | **SFTPA1B** | Q8IWL2 |
| **REG1A** | P05451 | **SDC4** | P31431 | **SFTPA2** | Q8IWL1 |
| **REG1B** | P48304 | **SDF2L1** | Q9HCN8 | **SFTPD** | P35247 |
| **REG3A** | Q06141 | **SECTM1** | Q8WVN6 | **SGCA** | Q16586 |
| **REG3G** | Q6UW15 | **SELE** | P16581 | **SGCE** | O43556 |
| **REG4** | Q9BYZ8 | **SELL** | P14151 | **SHH** | Q15465 |
| **RELT** | Q969Z4 | **SELP** | P16109 | **SHISA2** | Q6UWI4 |
| **RET** | P07949 | **SELPLG** | Q14242 | **SHISA3** | A0PJX4 |
| **RETN** | Q9HD89 | **SEMA3C** | Q99985 | **SHISA4** | Q96DD7 |
| **RETNLB** | Q9BQ08 | **SEMA3E** | O15041 | **SIDT2** | Q8NBJ9 |
| **RGMB** | Q6NW40 | **SEMA3F** | Q13275 | **SIGLEC10** | Q96LC7 |
| **RLN3** | Q8WXF3 | **SEMA3G** | Q9NS98 | **SIGLEC11** | Q96RL6 |
| **RNASE7** | Q9H1E1 | **SEMA4A** | Q9H3S1 | **SIGLEC15** | Q6ZMC9 |
| **RNFT2** | Q96EX2 | **SEMA4B** | Q9NPR2 | **SIGLEC5** | O15389 |
| **ROBO1** | Q9Y6N7 | **SEMA4D** | Q92854 | **SIGLEC6** | O43699 |
| **ROBO2** | Q9HCK4 | **SEMA4G** | Q9NTN9 | **SIGLEC7** | Q9Y286 |
| **ROBO4** | Q8WZ75 | **SEMA5A** | Q13591 | **SIGLEC8** | Q9NYZ4 |
| **ROR1** | Q01973 | **SEMA6A** | Q9H2E6 | **SIGLEC9** | Q9Y336 |
| **ROR2** | Q01974 | **SEMA6B** | Q9H3T3 | **SIL1** | Q9H173 |
| **RSPO3** | Q9BXY4 | **SEMA6C** | Q9H3T2 | **SIRPA** | P78324 |
| **RTN4R** | Q9BZR6 | **SEMA6D** | Q8NFY4 | **SIRPB2** | Q5JXA9 |
| **SAA1** | P0DJI8 | **SEMG1** | P04279 | **SIRPD** | Q9H106 |
| **SAA2** | P0DJI9 | **SEMG2** | Q02383 | **SIRPG** | Q9P1W8 |
| **SAA4** | P35542 | **SERPINA1** | P01009 | **SIT1** | Q9Y3P8 |

| | | | | | |
|---|---|---|---|---|---|
| **SLAMF1** | Q13291 | **SRGN** | P10124 | **TIMP2** | P16035 |
| **SLAMF6** | Q96DU3 | **SRPX** | P78539 | **TIMP4** | Q99727 |
| **SLAMF7** | Q9NQ25 | **SST** | P61278 | **TINAGL1** | Q9GZM7 |
| **SLAMF8** | Q9P0V8 | **STATH** | P02808 | **TLR1** | Q15399 |
| **SLC39A4** | Q6P5W5 | **STC1** | P52823 | **TLR2** | O60603 |
| **SLC39A6** | Q13433 | **STC2** | O76061 | **TLR3** | O15455 |
| **SLITRK1** | Q96PX8 | **SUSD1** | Q6UWL2 | **TLR4** | O00206 |
| **SLITRK3** | O94933 | **SUSD2** | Q9UGT4 | **TMC2** | Q8TDI7 |
| **SLITRK4** | Q8IW52 | **SUSD4** | Q5VX71 | **TMED1** | Q13445 |
| **SLITRK5** | O94991 | **TAC1** | P20366 | **TMEFF1** | Q8IYR6 |
| **SLITRK6** | Q9H5Y7 | **TAC3** | Q9UHF0 | **TMEFF2** | Q9UIK5 |
| **SLPI** | P03973 | **TAPBPL** | Q9BX59 | **TMEM108** | Q6UXF1 |
| **SLURP1** | P55000 | **TARM1** | B6A8C7 | **TMEM119** | Q4V9L6 |
| **SMCR7** | Q96C03 | **TCN1** | P20061 | **TMEM123** | Q8N131 |
| **SMOC1** | Q9H4F8 | **TCP11** | Q8WWU5 | **TMEM130** | Q8N3G9 |
| **SMOC2** | Q9H3U7 | **TCTN2** | Q96GX1 | **TMEM132D** | Q14C87 |
| **SMR3A** | Q99954 | **TCTN3** | Q6NUS6 | **TMEM154** | Q6P9G4 |
| **SMR3B** | P02814 | **TDGF1** | P13385 | **TMEM167A** | Q8TBQ9 |
| **SOST** | Q9BQB4 | **TECTB** | Q96PL2 | **TMEM190** | Q8WZ59 |
| **SOSTDC1** | Q6X4U4 | **TEK** | Q02763 | **TMEM207** | Q6UWW9 |
| **SPACA1** | Q9HBV2 | **TEX101** | Q9BY14 | **TMEM25** | Q86YD3 |
| **SPACA3** | Q8IXA5 | **TEX264** | Q9Y6I9 | **TMEM27** | Q9HBJ8 |
| **SPACA5** | Q96QH8 | **TF** | P02787 | **TMEM81** | Q6P7N7 |
| **SPAG11A** | Q6PDA7 | **TFF1** | P04155 | **TMEM98** | Q9Y2Y6 |
| **SPARC** | P09486 | **TFF2** | Q03403 | **TMEM9B** | Q9NQ34 |
| **SPARCL1** | Q14515 | **TFF3** | Q07654 | **TMIE** | Q8NEW7 |
| **SPESP1** | Q6UW49 | **TFPI** | P10646 | **TMIGD1** | Q6UXZ0 |
| **SPINK1** | P00995 | **TFPI2** | P48307 | **TMIGD2** | Q96BF3 |
| **SPINK14** | Q6IE38 | **TFRC** | P02786 | **TMPRSS11D** | O60235 |
| **SPINK2** | P20155 | **TGFA** | P01135 | **TMPRSS11F** | Q6ZWK6 |
| **SPINK6** | Q6UWN8 | **TGFB1** | P01137 | **TMUB1** | Q9BVT8 |
| **SPINK7** | P58062 | **TGFB2** | P61812 | **TMX4** | Q9H1E5 |
| **SPINK9** | Q5DT21 | **TGFB3** | P10600 | **TNFAIP6** | P98066 |
| **SPINLW1** | O95925 | **TGFBI** | Q15582 | **TNFRSF10A** | O00220 |
| **SPINT1** | O43278 | **TGFBR1** | P36897 | **TNFRSF10B** | O14763 |
| **SPINT2** | O43291 | **TGFBR2** | P37173 | **TNFRSF10C** | O14798 |
| **SPN** | P16150 | **TGFBR3** | Q03167 | **TNFRSF10D** | Q9UBN6 |
| **SPOCK1** | Q08629 | **TGOLN2** | O43493 | **TNFRSF11A** | Q9Y6Q6 |
| **SPOCK2** | Q92563 | **THBS4** | P35443 | **TNFRSF11B** | O00300 |
| **SPOCK3** | Q9BQ16 | **THPO** | P40225 | **TNFRSF12A** | Q9NP84 |
| **SPON1** | Q9HCB6 | **THY1** | P04216 | **TNFRSF13B** | O14836 |
| **SPON2** | Q9BUD6 | **TIE1** | P35590 | **TNFRSF13C** | Q96RJ3 |
| **SPP1** | P10451 | **TIGIT** | Q495A1 | **TNFRSF14** | Q92956 |
| **SPPL3** | Q8TCT6 | **TIMD4** | Q96H15 | **TNFRSF17** | Q02223 |
| **SPRY1** | O43609 | **TIMP1** | P01033 | **TNFRSF18** | Q9Y5U5 |

| | |
|---|---|
| **TNFRSF19** | Q9NS68 |
| **TNFRSF1A** | P19438 |
| **TNFRSF1B** | P20333 |
| **TNFRSF21** | O75509 |
| **TNFRSF25** | Q93038 |
| **TNFRSF4** | P43489 |
| **TNFRSF6B** | O95407 |
| **TNFRSF8** | P28908 |
| **TNFRSF9** | Q07011 |
| **TNFSF10** | P50591 |
| **TNFSF13** | O75888 |
| **TNFSF13B** | Q9Y275 |
| **TNFSF14** | O43557 |
| **TNFSF15** | O95150 |
| **TNFSF18** | Q9UNG2 |
| **TNFSF8** | P32971 |
| **TNFSF9** | P41273 |
| **TPBG** | Q13641 |
| **TPSAB1** | Q15661 |
| **TPST1** | O60507 |
| **TREM1** | Q9NP99 |
| **TREM2** | Q9NZC2 |
| **TREML1** | Q86YW5 |
| **TREML2** | Q5T2D2 |
| **TREML4** | Q6UXN2 |
| **TRH** | P20396 |
| **TSKU** | Q8WUA8 |
| **TSLP** | Q969D9 |
| **TTR** | P02766 |
| **TWSG1** | Q9GZX9 |
| **TXNDC12** | O95881 |
| **TXNDC15** | Q96J42 |
| **TYRO3** | Q06418 |
| **TYRP1** | P17643 |
| **UCN2** | Q96RP3 |
| **ULBP1** | Q9BZM6 |
| **ULBP2** | Q9BZM5 |
| **UNC5B** | Q8IZJ1 |
| **UNC5C** | O95185 |
| **UNC5D** | Q6UXZ4 |
| **UPK3BL** | B0FP48 |
| **UTS2** | O95399 |
| **UTS2D** | **Q765I0** |
| **VASN** | Q6EMK4 |
| **VCAM1** | P19320 |
| **VCAN** | P13611 |
| **VEGFA** | P15692 |
| **VEGFB** | P49765 |
| **VEGFC** | P49767 |
| **VIT** | Q6UXI7 |
| **VLDLR** | P98155 |
| **VMO1** | Q7Z5L0 |
| **VNN1** | O95497 |
| **VSIG1** | Q86XK7 |
| **VSIG10** | Q8N0Z9 |
| **VSIG2** | Q96IQ7 |
| **VSIG4** | Q9Y279 |
| **VSTM1** | Q6UX27 |
| **VSTM2A** | Q8TAG5 |
| **VSTM2L** | Q96N03 |
| **VTCN1** | Q7Z7D3 |
| **VTN** | P04004 |
| **VWC2** | Q2TAL6 |
| **WBP1** | Q96G27 |
| **WFDC10A** | Q9H1F0 |
| **WFDC11** | Q8NEX6 |
| **WFDC12** | Q8WWY7 |
| **WFDC2** | Q14508 |
| **WFIKKN2** | Q8TEU8 |
| **WIF1** | Q9Y5W5 |
| **WISP1** | O95388 |
| **WISP2** | O76076 |
| **WNT2** | P09544 |
| **XCL1** | P47992 |
| **XCL2** | Q9UBD3 |
| **XPNPEP2** | O43895 |
| **XXYLT1** | Q8NBI6 |
| **YME1L1** | Q96TA2 |
| **ZDHHC11** | Q9H8X9 |
| **ZG16** | O60844 |
| **ZG16B** | Q96DA0 |
| **ZPLD1** | Q8TCW7 |

**Supplementary Table 2 (associated to Figure 6).** Enriched GO biological process terms and corresponding p-values for the host genes targeted by the E3 proteins, split by HAdV species of the interacting E3 protein. NA indicates the absence of genes in a particular GO category, represented in grey color in Figure 6A.

| GO Term | | HAdV-A | HAdV-B | HAdV-D | HAdV-E | HAdV-F | New Term |
|---|---|---|---|---|---|---|---|
| GO:0051051 | negative regulation of transport | -0.75 | NA | -2.84 | -1.42 | -0.96 | Inhibition of transport process |
| GO:0050853 | B cell receptor signaling pathway | NA | NA | -2.79 | NA | NA | B cell receptor signaling |
| GO:0051248 | Negative regulation of protein metabolic process | -0.73 | NA | -2.74 | -1.39 | NA | Inhibition of protein metabolism |
| GO:0070663 | regulation of leukocyte proliferation | -0.65 | NA | -2.43 | -1.31 | -0.85 | Leukocyte proliferation |
| GO:0050863 | regulation of T cell activation | -0.57 | NA | -2.12 | -1.22 | -0.77 | T cell activation |
| GO:0071396 | cellular response to lipid | -0.80 | NA | -2.00 | -1.47 | -1.01 | Cellular response to lipids |
| GO:0050866 | negative regulation of cell activation | -0.83 | NA | -2.09 | -1.50 | -1.04 | Inhibition of cell activation |
| GO:0002244 | hematopoietic progenitor cell differentiation | NA | NA | -2.24 | NA | NA | Hematopoietic progenitor cell differentiation |
| GO:0050852 | T cell receptor signaling pathway | NA | NA | -2.24 | NA | NA | T cell receptor signaling |
| GO:0097028 | dendritic cell differentiation | -1.41 | NA | -2.32 | -2.11 | -1.63 | Dendritic cell differentiation |
| GO:0002820 | negative regulation of adaptive immune response | -1.51 | NA | -2.52 | -2.20 | NA | Inhibition of adaptive immune processes |
| GO:0002709 | regulation of T cell mediated immunity | -1.27 | NA | -2.03 | -1.96 | NA | T cell-mediated immunity |
| GO:0001906 | cell killing | -2.14 | -0.88 | -2.22 | -1.55 | NA | Cell killing |
| GO:0002228 | natural killer cell mediated immunity | -2.79 | -1.19 | -0.77 | -1.88 | NA | NK cell-mediated immunity |
| GO:0006950 | response to stress | -2.09 | -0.54 | -0.34 | -0.42 | -0.48 | Response to stress |
| GO:0032943 | mononuclear cell proliferation | -0.56 | NA | -2.06 | -1.20 | -0.75 | Mononuclear cell proliferation |
| GO:0051607 | defense response to virus | -0.83 | NA | -2.09 | -1.50 | NA | Antiviral response |
| GO:0050854 | regulation of antigen | NA | NA | -2.79 | NA | NA | Inhibition of |

| | | | | | | | |
|---|---|---|---|---|---|---|---|
| | receptor-mediated signaling pathway | | | | | | antigen-receptor signaling |
| GO:0002683 | negative regulation of immune system process | -0.68 | NA | -2.54 | -1.34 | -0.88 | Inhibition of immune system processes |
| GO:0072511 | divalent inorganic cation transport | -0.96 | NA | -2.49 | -1.64 | -1.18 | Inorganic cation transport |
| GO:0002764 | immune response-regulating signaling pathway | -0.61 | NA | -2.27 | -1.26 | -0.81 | Regulation of immune signaling pathways |
| GO:0002703 | regulation of leukocyte mediated immunity | -0.89 | NA | -2.25 | -1.56 | NA | Regulation of immune signaling pathways |
| GO:0051348 | negative regulation of transferase activity | NA | NA | -2.03 | NA | NA | Inhibition of transferase activity |

**Supplementary Table 3 (associated to Figure 6).** Disease terms from the DisGeNET database were manually collapsed to create curated terms shown in Figure 6. Table shows which disease terms were grouped together to generate the terms used for the disease association network.

| Disease association, curated term | Disease association, DisGeNET term |
|---|---|
| Bacterial and parasitic diseases | Bacterial infections and mycoses; Parasitic diseases |
| Viral infection | Virus diseases |
| Cancer | Neoplasms |
| Nervous diseases and neurological disorders | Nervous system diseases, Behavior and behavior mechanisms |
| Eye disease | Eye diseases |
| Gastrointestinal diseases | Digestive system diseases, Male urogenital diseases, Female urogenital diseases and pregnancy complications |
| Respiratory track disease | Respiratory track diseases, Otorhinolaryngologic diseases |
| Immune disease | Immune system diseases, Hemic and lymphatic diseases |
| Other | Musculoskeletal diseases; Cardiovascular diseases; Stomatognathic diseases; Congenital, hereditary and neonatal diseases and abnormalities; Skin and connective tissue diseases; Nutritional and metabolic diseases; Endocrine system diseases; Disorders of environmental origin; Animal diseases; Pathological conditions, signs and symptoms; Occupational diseases; Chemically-induced disorders; Wounds and injuries. |
